# Mitigating effects of Jiawei Chaihu Shugan decoction on necroptosis and inflammation of hippocampal neurons in epileptic mice

**DOI:** 10.1038/s41598-025-89275-8

**Published:** 2025-02-07

**Authors:** Qin Wang, Baijun Qin, Han Yu, Jiawei Zeng, Jingjing Fan, Qiong Wu, Rong Zeng, Haichun Yu, Xian Zhang, Mingfen Li, Yanying Zhou, Limei Diao

**Affiliations:** 1 The First Clinical School of Medicine, Guangxi University of Chinese Medicine, 179 Mingxiu East Road, Nanning, 530001 Guangxi China; 2 Department of Neurology, The First Affiliated Hospital of Guangxi University of Chinese Medicine, No. 89-9 Dongge Road, Qingxiu District, Nanning, 530023 Guangxi China; 3 Department of Gastroenterology, Chongqing City Hospital of Traditional Chinese Medicine, No. 6, Panxi seventh branch road, Jiangbei District, Chongqing, 400021 China; 4 Harbin Medical University, 157 Baojian Road, Nangang District, Harbin, 150081 Heilongjiang China; 5 Xinyang Central Hospital, Xinyang, 464000 Henan China; 6 Qinzhou Maternal and Child Health Hospital (Qinzhou Red Cross Hospital), No.1 Anzhou Avenue, Qinzhou City, Guangxi Zhuang Autonomous Region China; 7 Guangxi Technological College of Machinery and Electricity, Nanning, 530007 Guangxi China; 8 Guangxi Zhuang Autonomous Region Brain Hospital, Liuzhou, 545005 Guangxi China; 9 Department of Clinical Laboratory, The First Affiliated Hospital of Guangxi University of Chinese Medicine, Nanning, 530023 Guangxi China

**Keywords:** Jiawei Chaihu Shugan decoction, Epilepsy, Hippocampal neurons, Necroptosis, Inflammation, Signaling pathway, Molecular biology, Neuroscience, Biomarkers, Neurology, Pathogenesis

## Abstract

Jiawei Chaihu Shugan decoction (JWCHSGD) is a traditional Chinese medicine well-known for its beneficial effects in treating epilepsy (*Xianzheng* in ancient Chinese), but the molecular mechanism of its action remains unclear. To investigate the molecular mechanism of JWCHSGD’s prevention of epilepsy-mediated neuron from necroptosis and inflammation via the circRNA-Csnk1g3/Csnk1g3-85aa/ CK1γ3/TNF-α signal pathway. In vitro, murine neuronal HT22 cells were treated in six groups: control, model, carbamazepine, and three JWCHSGD doses (high, medium, low). Viability and apoptosis were assessed via CCK-8 and flow cytometry. In vivo, 60 C57BL/6J mice were divided into six groups: control, model, carbamazepine, JWCHSGD, JWCHSGD + Sh Circ_Csnk1g3, and JWCHSGD + Sh NC. An epilepsy model was induced, and treatments were administered for two weeks. Outcomes included EEG, hippocampal histopathology, apoptosis (TUNEL), and mRNA/protein expression of key pathway markers. In HT22 cells, the model group showed reduced viability, increased apoptosis, and elevated mRNA/protein levels of Csnk1g3-85aa, RIP1, RIP3, MLKL, TNF-α, IL-6, and IL-1β (*P* < 0.05). JWCHSGD and carbamazepine increased viability and decreased apoptosis, reversing these molecular changes (*P* < 0.05). In mice, the model group had heightened epileptic discharges, neuronal damage, and apoptosis, along with increased expression of the same markers (*P* < 0.05). JWCHSGD and carbamazepine mitigated these effects (*P* < 0.05). JWCHSGD reduces epileptic events by regulating the circRNA-Csnk1g3/Csnk1g3-85aa/CK1γ3/TNF-α signaling pathway, impacting necroptosis and inflammation in hippocampal neurons and HT22 cells.

**Supplementary Information:**

The online version contains supplementary material available at 10.1038/s41598-025-89275-8.

## Introduction

Epilepsy is a neurological disease manifesting with repeated abnormal discharges of brain neurons and disruption of network structure^1,2^. According to recent reports, the prevalence of epilepsy in China has reached 35.0 per 100,000 people annually, making it the second largest category of neurological diseases in clinical practice^3,4^. Various types of epilepsy are often accompanied by pathological changes such as necroptosis, inflammatory responses, oxidative stress, mossy fiber sprouting, and synaptic remodeling^5^. There are no targeted treatments available for these pathological changes. Currently, even with regular antiepileptic drug therapy, about 30% of patients may develop refractory epilepsy^6^. Therefore, it is crucial to explore the pathogenesis of epilepsy and develop better treatment strategies.

Traditional Chinese Medicine (TCM) believes that treating epilepsy by focusing on the liver is highly effective^7^. The Jiawei Chaihu Shugan decoction (JWCHSGD) is derived from the Chaihu Shugan decoction recorded in the *Jingyue Quanshu*, and has been modified to soothe the liver, regulate Qi, and alleviate depression, thereby treating both the root cause and symptoms of epilepsy^8^. Long-term clinical observations have shown that JWCHSGD has good clinical efficacy in reducing the frequency of epileptic seizures^9^. Animal experiments revealed that JWCHSGD can reduce epileptic waves in the electroencephalograms (EEGs) of epileptic mice and improve their cognitive function. This may be achieved by regulating pathways such as lncRNA-UCA1/miR-187/MAPK8, miR-187/Wnt/β-catenin, miR-204/AKT/mTOR/P70S6K, or by inhibiting the expression of autophagy-related protein 7 (ATG7) and microtubule-associated protein light chain 3 (LC3). This achieves the purpose of treating epilepsy by protecting brain neurons through reducing autophagy and apoptosis in the hippocampal neurons of epileptic mice^8–13^.

To further explore the mechanism of action of this decoction, we constructed an acute epilepsy mouse model and performed second-generation high-throughput sequencing. Compared with normal mice, we found that circRNA-Csnk1g3 was upregulated in the hippocampal tissues of epileptic mice. This result was corroborated by quantitative real-time PCR (qRT-PCR)^14^. Using the IRESfinder software and ORF prediction websites, we discovered that circRNA-Csnk1g3 contains an internal ribosome entry site (IRES) and an open reading frame (ORF) located at 247–504 bp in the circular RNA, comprising a 258 bp segment that encodes an 85 amino acid polypeptide, which we named ‘Csnk1g3-85aa’. This suggests that circRNA-Csnk1g3 has translation potential and may be involved in the pathogenesis of epilepsy by encoding polypeptide segments such as Csnk1g3- 85aa^15^.

Bioinformatics analysis and Circbase database searches revealed that the potential parental protein of circRNA-Csnk1g3 was CK1γ3. Previous studies indicated that phosphorylation of CK1γ3 can activate relevant apoptotic proteins in the TNF-α necroptosis signaling pathway, such as RIP1, RIP3, and MLKL, inducing necroptosis of hippocampal cells and leading to epilepsy^16^. Combining network pharmacology analysis, we also found that the TNF necroptosis signaling pathway is one of the key pathways through which JWCHSGD’s anti-epileptic activity is mediated, with TNF-α, IL-1β, and IL-6 being the core targets of this compound formula^17,18^.

**Fig. 1:**
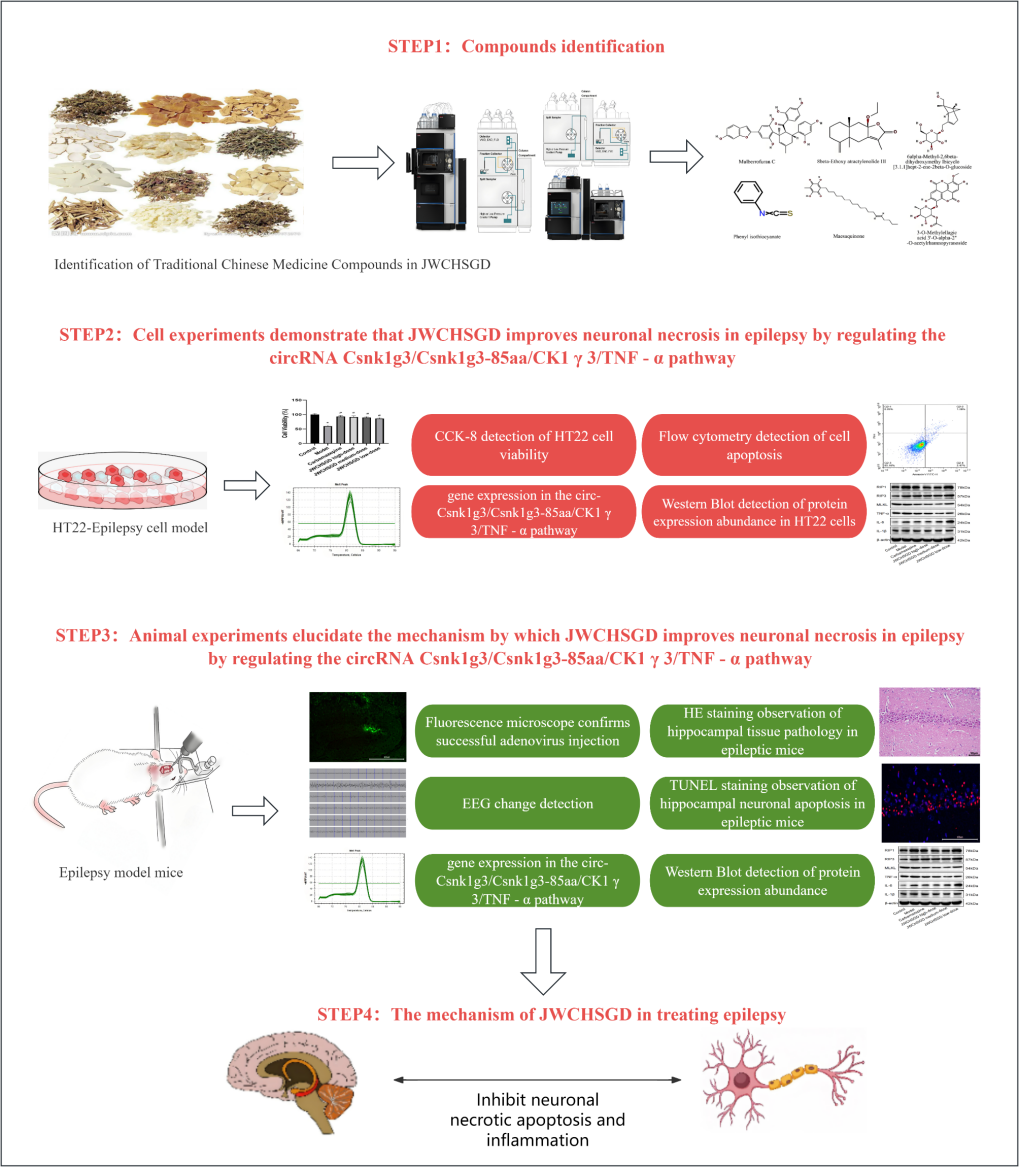
Overview of the key procedures in this study.

Our purpose here was to determine whether JWCHSGD could reduce hippocampal neuronal necroptosis and inflammatory responses and control epileptic seizures by regulating the circ-Csnk1g3/Csnk1g3-85aa/CK1γ3/TNF signaling pathway through in vivo and in vitro experiments. The key procedure of this research is outlined in the figure below (Fig. 1).

## Materials and methods

### Animals, cells, and reagents

Twenty-one 7-week-old male SPF SD rats were obtained from Beijing Huafukang Bioscience Co., Ltd (License No. SYXK (Gui) 2009-0001) and used to prepare drug-containing serum. The HT22 mouse hippocampal neuronal cell line was purchased from Procell (CL-0697, Wuhan, China). Sixty 6-wk-old male SPF C57BL/6J mice were purchased from Beijing Sibeifu Biotechnology Co., Ltd (License No. SYXK (Gui) 2009-0001). The following items were obtained from the indicated supplier: complete DMEM (KGM12800S, Kaiji Bio), incomplete DMEM (KGM12800N, Kaiji Bio), FBS (10099-141, Gibco), Lipofectamine 3000 transfection reagent (L3000015, Invitrogen), OptiMEM (31985-062, Gibco), trypsin-EDTA digestion solution (T1300, Solarbio), 1× PBS (0.01 M, pH7.4) (KGB5001, Kaiji Bio), Trizon reagent (CW0580S, CWBIO), ultrapure RNA extraction kit (CW0581M, CWBIO), HiScript II Q RT SuperMix for qPCR (+ gDNA wiper) (R223-01, Vazyme), ChamQ Universal SYBR qPCR master mix (Q711-02, Vazyme), RIPA lysis buffer (C1053, Beijing Prumeikai Gene Technology Co., Ltd.), BCA protein quantitation kit (E-BC-K318-M, Elabscience), sodium dodecyl sulfate (SDS) (151-21-3, Xilong Science Co., Ltd.), PVDF membranes (IPVH00010, Millipore), blocking-specific powdered skim milk (P1622, Beijing Prumeikai Gene Technology Co., Ltd.), DAB staining kit (CW0125M, CWBIO), xylene (33535, Xilong Science Co., Ltd.), anhydrous ethanol (32061, Xilong Science Co., Ltd.), 95% ethanol (32061, Xilong Science Co., Ltd.), TUNEL detection kit (red) (C1090, Beyotime), hematoxylin stain (ZLI-9610, Zhongshan Golden Bridge), eosin stain (G1100, Solarbio), hematoxylin bluing solution (G1040, Servicebio), ultra-clean mounting medium (YZB, BASO). The following antibodies were obtained from the indicated suppliers: internal control primary antibody, mouse anti-β-actin (HC201, TransGen Biotech); target primary antibodies: rabbit anti-Csnk1g3-85aa synthesized by Hangzhou Huaan Biotechnology Co., Ltd, rabbit anti-CK1γ3 (AF9034, Affinity), rabbit anti-RIP1 (13176-AP, Proteintech), rabbit anti-RIP3 (AF7942, Affinity), rabbit anti-MLKL (OM155258, OmnimAbs), mouse anti-TNF-α (60291-1-lg, Proteintech), rabbit anti-IL-1β (sc-32294, Santa Cruz Biotechnology), rabbit anti-IL-6 (DF6087, Affinity); secondary antibodies: HRP-conjugated goat anti-rabbit IgG (H + L) (GB23303, Servicebio) and HRP-conjugated goat anti-mouse IgG (H + L) (GB23301, Servicebio). The Circ Csnk1g3 and its NC control virus and plasmids were provided by Jiangxi Zhonghongboyuan Co., Ltd.

Experimental animals were housed in the Animal Center of the Guangxi Key Laboratory of Molecular Medicine in Prevention and Treatment of Major Diseases by Traditional Chinese Medicine. Animals were in separated cages, 5 mice per cage, with SPF conditions (temperature 20–26 °C, relative humidity 40–70%), 12-h light-dark cycle, and ad libitum food and water. Cardiac blood collection was performed after euthanizing a mixture of Zoletil and Xylazine anesthetic agent intraperitoneally. The anesthetic used was a mixture of Zoletil and Xylazine: a solution was prepared with Zoletil at 6 mg/ml and Xylazine at 1.786 mg/ml, administered intraperitoneally at a dose of 10 ml/kg. After intraperitoneal injection, cardiac blood collection was performed to induce euthanasia. The mouse was placed in a supine position and fixed. The heartbeat was palpated between the 3rd and 4th ribs on the left side to locate the area where it was strongest. A syringe with a 4–5 gauge needle was then inserted into the heart at a 45-degree angle at this point. Once the needle was correctly positioned, blood flowed naturally into the syringe due to the pumping action of the heart. The mouse was confirmed dead when the heart stopped beating and blood flow ceased. The procedures and related technical details of the experiments have been reviewed and approved by the Biosafety Review Committee and the Animal Ethics Committee of Guangxi University of Chinese Medicine (Approval No. DW20231016-225).

### Apparatus

CO₂ incubator (BPN-80CW, Shanghai Yiheng Scientific Instrument Co., Ltd.), inverted fluorescence microscope (MF53, Guangzhou Mingmei Optoelectronics Co., Ltd.), clean bench (BBS-SDC, Biobase), centrifuge (TD4A, Changsha Yingtai Instrument Co.), three-gas incubator (YCP-10 S, Changsha Huaxi Electronic Technology Co.), PCR amplifier (TC-EA, Hangzhou Borui Technology Co., Ltd.), fluorescence PCR instrument (CFX Connect™ Real-Time, Bio-Rad Life Science Products, Shanghai), ultra-sensitive chemiluminescence imaging system (ChemiDoc™ XRS+, Bio-Rad Life Science Products, Shanghai), fully automated microplate reader (SuPerMax 3100, Shanghai Flash), microplate reader (WD-2012B, Beijing Liu Yi), protein electrophoresis system (DYY-6 C, Beijing Liu Yi Instrument Factory), tissue dehydration machine (KD-TS3S1, Jinhua Kedi Instrument & Equipment Co.), paraffin embedding machine (HistoCore Arcadia, Leica), microtome (HistoCore Biocut, Leica), slide warmer (Leica HI1210, Leica), fluorescence microscope (BX53, Olympus), brain stereotaxic injection instrument (Model H1829401-002, Ruowood, Shenzhen, China).

### Source, composition, and preparation of the JWCHSGD decoction

The herb names listed below have been verified with MPNS (http://mpns.kew.org). JWCHSGD was prepared using the following ingredients: *Bupleurum chinense* DC. (Bei chai hu) 15 g (batch number 20220101, part used: dried root); *Paeonia lactiflora* Pall. (Bai shao) 15 g (batch number 20220201, part used: dried root); *Fructus Aurantii* (Zhi ke) 10 g (stir-fried with bran, batch number 220114, part used: dried root); *Glycyrrhiza uralensis* (Zhi gan cao) 6 g (batch number: 2202113, part used: dried root or rhizome); Ligusticum chuanxiong (Chuan xiong) 10 g (batch number: 20220202, part used: dried rhizome); *Cyperus rotundus* (Xiang fu) 10 g (batch number: 20220201, part used: dried rhizome); *Citri reticulatae Pericarpium* (Chen pi), 9 g (stir-fried with vinegar, batch number: 22022202, part used: dried fruit peel), *Fritillaria verticillata* (Zhe bei mu), 15 g, (batch number: 22020201, part used: dried bulb); Ostrea gigas (Sheng mu li), 30 g, (batch number: 220100803, part used: oyster shell); *Uncaria rhynchophylla* (Gou teng), 10 g, (batch number: 20220201, part used: hooked stems and branches); *Scolopendra* (Wu gong), 1 centipede, (batch number: 02202088, part used: dried body of centipede). All medicinal materials were procured from Guangxi Xianju Traditional Chinese Medicine Technology Co., Ltd. and were authenticated as genuine by Associate Professor Luo Dan of Guangxi University of Traditional Chinese Medicine, in accordance with the 2020 edition of the Chinese Pharmacopoeia. The decoction was prepared using the automated decoction machine of the First Affiliated Hospital of Guangxi University of Chinese Medicine. The final concentration was 500 mg/mL. Carbamazepine tablets (batch number H11022279, 200 mg/tablet) were from Novartis Pharmaceuticals Ltd.

### Liquid chromatography/tandem mass spectrometry (LC/MS-MS) determination of active ingredients in JWCHSGD

A sample of the JWCHSGD suspension (200 µL) was placed into an Eppendorf tube, vortexed 30 s, and then centrifuged (20 °C, 17,000*g*, 15 min). An aliquot of the supernatant (100 µL) was transferred into an insert tube and analyzed by LC-MS/MS with the following conditions: (1) mobile phase (a) column temperature = 40 °C and injection volume = 3 µL; (b) positive ion mode, A = 0.1% formic acid in water, B = 0.1% formic acid in acetonitrile; (c) negative ion mode, A = 2 mM ammonium acetate in water, B = acetonitrile. (2) Mass spectrometry using an AB 5600 triple TOF mass spectrometer with Analyst TF 1.7 software (AB Sciex) and IDA (information-dependent acquisition) function. During each data-acquisition cycle, molecular ions with the peak intensity > 100 were selected for acquiring MS/MS data.

### Preparation of drug-containing serum and magnesium-free extracellular medium

Twenty-one SD rats were arranged in three groups of seven rats each. Each group was administered an equal volume of saline, 30 mg/kg carbamazepine suspension, or 10 mL/kg JWCHSGD via gavage once daily for one week. Blood samples were taken, allowed to clot, and centrifuged to separate serum. The concentrations of drug-containing serum were prepared as follows: (1) control serum: incomplete DMEM with 5%, 7.5%, 10%, 15%, 20%, and 30% serum from saline-treated rats; (2) carbamazepine-containing serum: incomplete DMEM with 5%, 7.5%, 10%, 15%, 20%, and 30% serum from carbamazepine-treated rats; and (3) JWCHSGD-containing serum: incomplete DMEM with 5%, 7.5%, 10%, 15%, 20%, and 30% serum from JWCHSGD-treated rats.

Magnesium-free extracellular medium: 2 mM CaCl₂, 145 mM NaCl, 0.002 mM glycine, 10 mM glucose, 2.5 mM KCl, and 10 mM HEPES, pH 7.4.

### CCK-8 assay

**Fig. 2:**
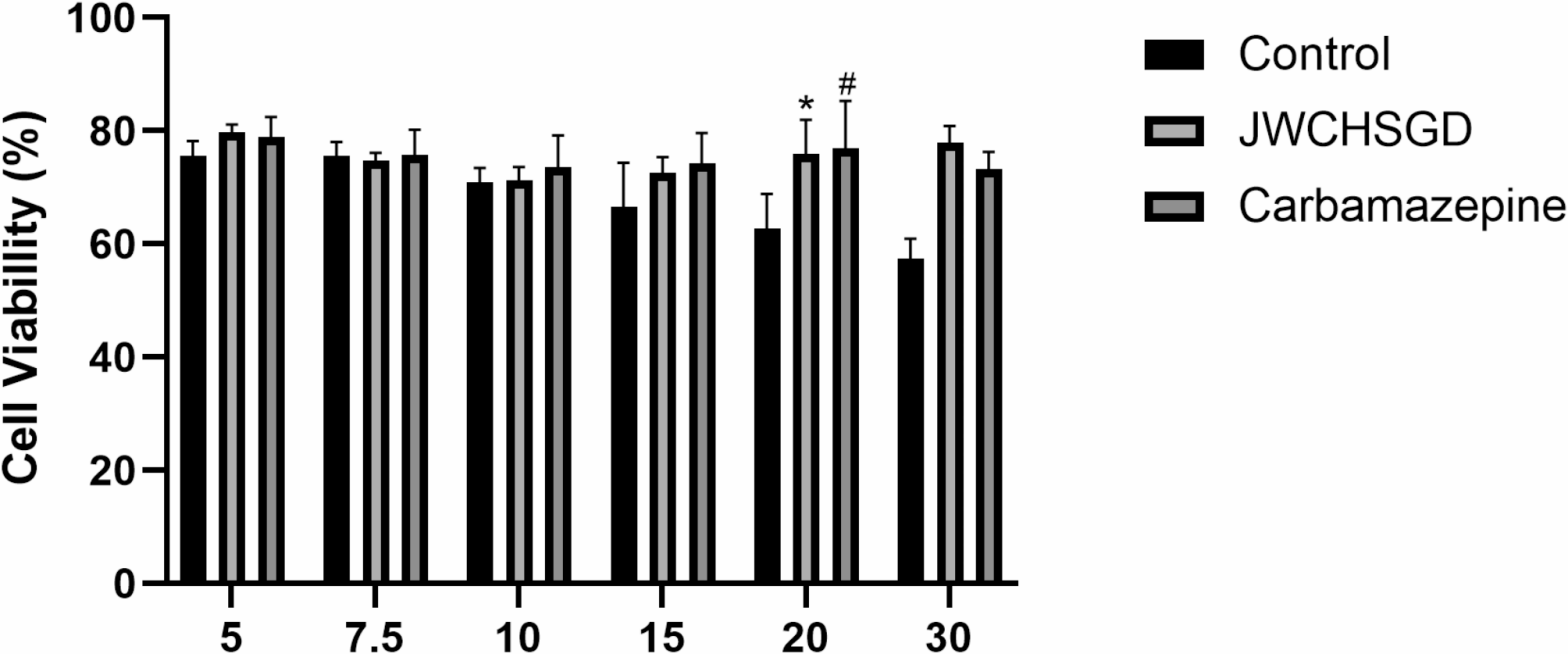
CCK-8 viability assay for dose-response of drug-containing serum. (*P < 0.05) JWCHSGD vs. control; (^#^*P* < 0.05) carbamazepine vs. control.

Different concentrations of serum were mixed with cultures of HT22 neuronal cells and incubated for 24, 48, and 72 h. After 24 h of treatment in a 96-well plate, the medium was replaced with fresh medium, and 10 µL of CCK-8 reagent was added to each well. The plate was incubated for 2 h at 37 °C, and the absorbance at 450 nm was measured using a microplate reader. The results showed that the drug-containing serum concentration gradient with 20% as the baseline was statistically significant. The results were used to select the following concentrations for subsequent experiments: control serum at 20%, carbamazepine-containing serum at 20%, and JWCHSGD-containing serum at 20%, 15%, and 10% (Fig. 2). The groups administered 20%, 15%, and 10% JWCHSGD-containing serum were named high-, medium-, and low-dose.

Based on the CCK results, it was observed that the serum control itself had an impact on the cells, and as the concentration was increased, the cell viability in the control group significantly decreased. This might be due to certain proteins, microorganisms, or other bioactive substances in the serum at high concentrations that negatively affected cell viability. Alternatively, some components of the serum at high concentrations may interfere with the interaction between the CCK-8 reagent and cellular metabolic activity, thereby reducing absorbance readings (which reflect cell viability). Therefore, for the CCK viability assay, we tested a range of serum concentrations to determine its effect on the assay and optimize the serum concentration.

### Cell culture, drug administration, and model establishment

HT22 cells were cultured for 24 h in 7 cm dishes, 6-well plates, and 12-well plates in a 37 °C, 5% CO_2_ incubator. Except for the control group, cells were incubated with magnesium-free extracellular medium for three hours to establish the model. Afterward, the corresponding drug-containing serum (as selected from “CCK-8 assay”) was added. Cells were collected 24 h later for analysis.

### FITC-labelled annexin-V assay for apoptosis

Suspensions of 10^6^ cells of each group were harvested, washed with PBS, suspended in 300 µL of pre-chilled annexin V-FITC binding reagent, and 5 µL of FITC-labeled annexin V and 10 µL of propidium iodide (PI) were added to each sample. The reactions were incubated at RT in the dark. Flow cytometry was used for detection and analysis.

### Animal groups, model establishment, and drug administration

After one week adaptation, mice were randomly arranged into six groups: (1) control, (2) model, (3) carbamazepine, (4) JWCHSGD (JWCHSGD), (5) JWCHSGD + Sh Circ_Csnk1g3 (knockdown of Circ_Csnk1g3 gene expression), and (6) JWCHSGD + Sh NC group (adenovirus empty vector group), with ten mice per group. Except for the control mice, all other mice were injected intraperitoneally with pilocarpine hydrochloride (180 mg/kg) to establish the model. Behavior was observed according to the classical Racine (1972) standard^19^ for seizures in experimental animals. Thirty minutes after injection, the mice were monitored for seizures of grade 4 or higher. If a mouse did not reach grade 4, additional injections of 1/3 of the original dose of pilocarpine hydrochloride were administered every 15 min, up to two additional injections. Mice that did not reach grade 4 seizures after these injections were considered to have failed model establishment and were excluded from further behavioral observation and index detection. After one hour of seizure activity, an appropriate dose of atropine sulfate was injected intraperitoneally to terminate the seizures^20^. During the modeling period, EEG was monitored to record the onset time, duration, and intensity of seizures in, excluding those without spontaneous recurrent seizures. All mice in this experiment were successfully modeled, and those not meeting the modeling criteria were excluded.

After model establishment, the control and model groups were gavaged with equal volumes of saline. The carbamazepine group received 30 mg/kg carbamazepine suspension by gavage. The JWCHSGD group, JWCHSGD + Sh Circ_Csnk1g3 group, and JWCHSGD + Sh NC group were treated with JWCHSGD at a dose of 19.52 g/kg by oral gavage once daily for 2 weeks. The dosage was calculated based on the *Modern Medical Experimental Animal Science*^21^ and referenced from animal experiments previously published by our research group^22^.

### Stereotaxic injection

Four days before the termination of drug administration, the mice were anesthetized, and the top of their heads were shaved and prepared. The mice were fixed on the stereotaxic apparatus using ear bars, and a 1.5 cm incision was made along the midline of the scalp. The lambda and bregma points were identified, and the head was leveled in the anterior-posterior and medial-lateral directions. Using an experimental animal brain atlas, the hippocampal coordinates were determined and marked. The stereotaxic parameters for the hippocampal region were selected based on the mouse stereotaxic atlas: using the intersection of the coronal suture and sagittal suture (bregma point) as the zero point, 2.0 mm posterior, 2.0 mm lateral, and 2.0 mm deep to the subdural space^23^. After positioning, a burr hole was created at the designated location on the skull using a dental drill gently to avoid damaging the dura mater and surface blood vessels of the brain tissue. A microinjection syringe was filled with 15 µL of Circ_Csnk1g3 interference adenovirus, ensuring that all air bubbles were expelled. The syringe was connected to a microinjection pump, and the manual push-button on the pump was used to remove any remaining air from the syringe needle. The appropriate volume and speed were set on the injection pump, and the total injection time was 20 min. The microinjection pump was started, and the injection was carried out, with the needle left in place for five minutes after completion.

### EEG measurements

After anesthetizing the mice, they were secured in the stereotaxic apparatus, and the skull was fully exposed. A craniotome was used to create windows on the left and right temporal regions, approximately 2 × 2 mm square. A small amount of saline was applied to keep the exposed brain tissue moist. Electrodes were inserted into the temporal bone surface and connected to electrodes on the same side of the ear. Once the recordings had stabilized, the EEGs were obtained (*n* = 10, repeated three times). The NCERP5.01 data analysis software was used to record cortical EEGs, with a paper speed of 3 cm/s and sensitivity 100 µV/cm. EEG abnormalities were classified as diffuse, focal, paroxysmal rhythmic wave abnormalities, and spike-wave patterns according to standard diagnostic criteria^24^.

### Hematoxylin and eosin staining (H & E)

The brain tissue, which was fixed with 4% paraformaldehyde underwent dehydration overnight, embedding in paraffin, sectioning, baking, deparaffinization, rehydration, and H & E staining sequentially. After mounting, the hippocampal neurons were examined with a visible-light microscope for morphology, number, and distribution. Digital images were obtained for analysis.

### TUNEL staining

Paraffin sections of hippocampal tissue were deparaffinized, dehydrated, and digested with protease. The samples were washed three times with PBS and 50 µL of TUNEL reaction mixture was added to the specimens and incubated in the dark at 37 °C for 1 h. The specimens were rinsed with PBS and nuclei were stained with DAPI in the dark at 15–25 °C for 5 min. After PBS washing, excess liquid was absorbed with tissue paper, and the slides were mounted with an anti-fade mounting medium. Digital images were obtained with a digital camera on a fluorescence microscope (*n* = 10, repeated three times).

### RT-PCR

Total RNA was purified from hippocampal tissue (*n* = 10, repeated three times) and HT22 cells using TRIzol. RNA concentration (400–800 ng/µL) and purity (A260/A280 ratio: 1.8-2.0) were assessed. Using reference gene primers and target gene primers along with reverse transcriptase, cDNA was synthesized. Real-time quantitative PCR (RT-qPCR) was performed with the following parameters: pre-denaturation at 95 °C for 10 min; denaturation at 95 °C for 10 s; annealing at the temperatures listed in Table 2 for 30 s; extension at 72 °C for 30 s; 40 cycles. Beta-actin was used for normalization and relative target gene expression was calculated using the 2-△△Ct method. The primers are listed in Table 1.

**Table 1:** Primer sequences.

Primer	Primer sequence (5′–3′)
β-actin F	AGGGAAATCGTGCGTGAC
β-actin R	CATACCCAAGAAGGAAGGCT
Circ-Csnk1g3 F	TCGGATGTGGCAATTTTGG
Circ-Csnk1g3 R	AACGTCAGCGGATAGTCAGTCA
Csnk1g3-85aa F	CAAATCAGATGATAGAATGGCAC
Csnk1g3-85aa R	CCAGATGAAGAAGACCCAGTTCC
RIP1 F	TGTAGAAGAGGATGTGGCAAGTTTA
RIP1 R	GCGATCCAGGTTGTTCTGAATTT
RIP3 F	GTTTCTAAAATGCTGGACCGC
RIP3 R	ATGTGGAATCTGAGGAGTGCC
MLKL F	GGAAAGATCCCATTTGAAGGCTGT
MLKL R	TTTCCCGCAACAACTCAGGG
TNF-α F	CAGGCGGTGCCTATGTCTC
TNF-α R	CGATCACCCCGAAGTTCAGTAG
IL-1β F	GAAATGCCACCTTTTGACAGTG
IL-1β R	TGGATGCTCTCATCAGGACAG
IL-6 F	TCCGGAGAGGAGACTTCACA
IL-6 R	TTGCCATTGCACAACTCTTTTC

### Western immunoblot protein assays

Total protein was purified from hippocampal tissue (*n* = 10, repeated three times) and HT22 cells using RIPA lysis buffer. The samples were centrifuged at 12,000 rpm for 10 min at 4 °C, and the supernatants were collected. Protein concentrations were determined by BCA assay. Following protein denaturation, SDS-PAGE was conducted, and proteins were electro-transferred to PVDF membranes at 300 mA for 1 h. The PVDF blots were blocked with skim milk, incubated with primary Abs at 4 °C overnight, then with secondary Abs at RT for 2 h the next day. The blots were then treated with chemiluminescence reagent and developed using a high-sensitivity chemiluminescence imaging system. Beta-actin served as the housekeeping control for statistical analysis and to calculate relative expressions of target proteins. The Abs and dilution factors are shown in Table 2.

**Table 2:** Western blot antibodies and dilution factors.

Antibody	Dilution factor
Anti-β-Actin (HC201, TransGen Biotech)	1:2000
IgG (H + L) (GB23301, Servicebio)	1:2000
Rabbit Anti-Csnk1g3-85aa (synthesized by Hangzhou Huaan Biotechnology Co., Ltd)	1:1000
Rabbit Anti-CK1γ3 (Affinity AF9034)	1:1000
Rabbit Anti-RIP1 (Proteintech 13176-1-AP)	1:3000
Rabbit Anti-RIP3 (Affinity af7942)	1:1000
Rabbit Anti-MLKL (OmnimAbs OM155258)	1:500
Mouse Anti-TNF- α (proteintech 60291-1-Ig)	1:4000
Rabbit Anti-IL-1β (Santa Cruz SC-32294)	1:1000
Rabbit Anti-IL-6 (Affinity df6087)	1:1000

### Statistical analyses

GraphPad Prism (ver. 9.5.1, GraphPad Software, San Diego, CA, US) and SPSS (ver. 26.0, IBM, NY, US) were used for graphs and statistical analyses. Quantitative results are expressed as means ± standard deviation ($\:\stackrel{-}{x}$± sd). Data distributions were tested for normality and homogeneity of variance. Multiple group comparisons were done by one-way ANOVA. Statistical significance was set at α = 0.05, with *P* < 0.05 considered significant. When necessary, Bonferroni post-hoc tests were used for multiple comparisons.

## Results

### Identification of compounds in JWCHSGD by LC-MS/MS

**Fig. 3:**
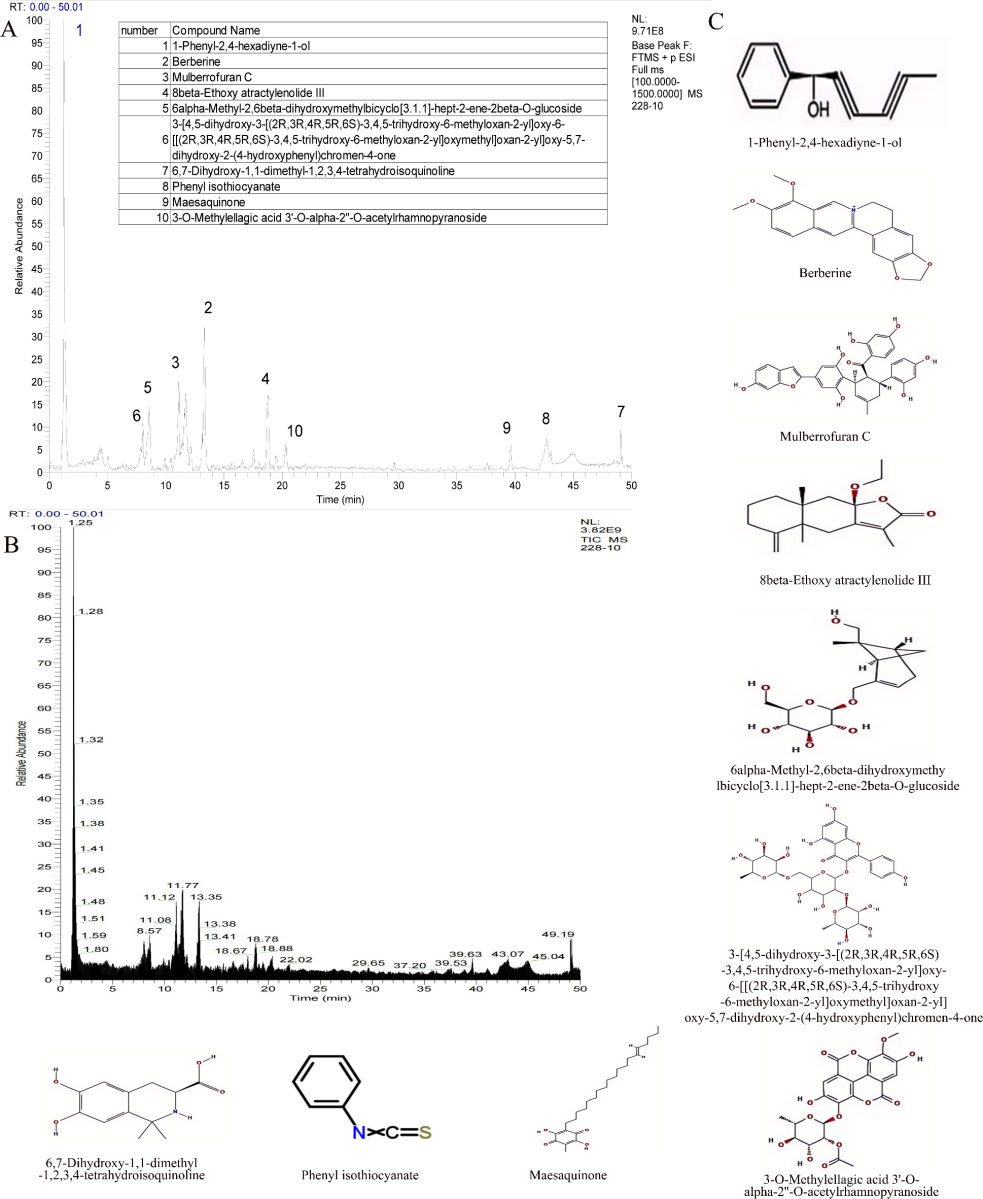
LC-MS/MS identification of major compounds in JWCHSGD. (**A**) Top ten compounds by relative abundance and corresponding peak markers on the total ion chromatogram. (**B**) Total ion chromatogram. (**C**) Molecular formulas of the top ten compounds.

The main chemical components of JWCHSGD were identified by LC-MS. The total-ion chromatograms (TICs) showed the following major components present in JWCHSGD: 1-phenyl-2,4-hexadiyne-1-ol; berberine; mulberrofuran C; 8-beta-ethoxy atractylenolide III; 6,7-dihydroxy-1,1-dimethyl-1,2,3,4-tetrahydroisoquinoline; phenyl isothiocyanate; and maesaquinone (Fig. 3).

### CCK-8 assay of HT22 cell viability

**Fig. 4:**
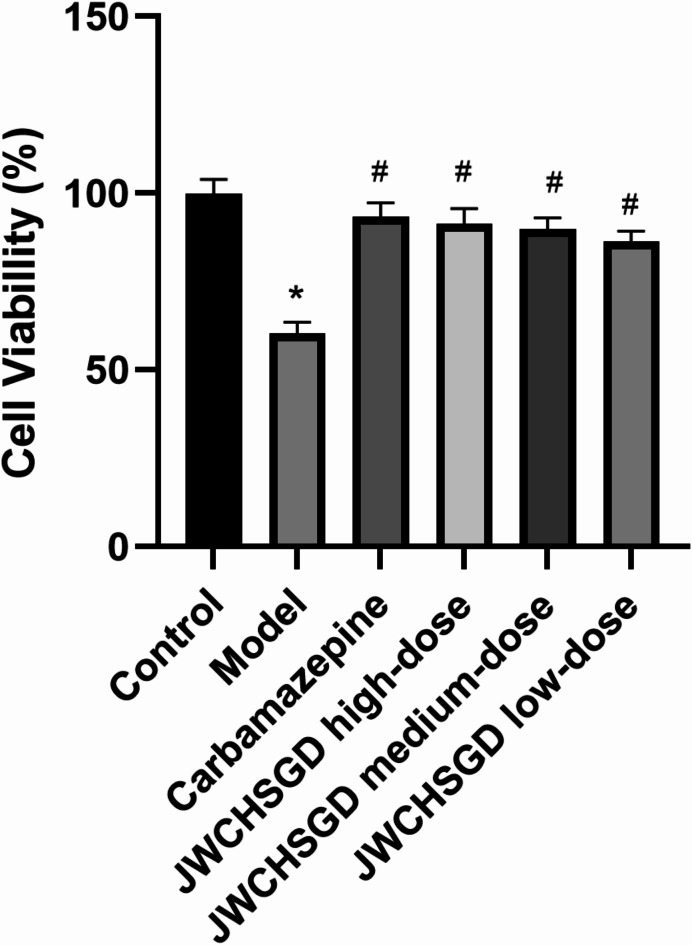
CCK-8 assay of HT22 cell viability. (*P < 0.05) model vs. control; (^#^*P* < 0.05) treatment vs. model.

The assay results indicated that the viability of HT22 cells in the model group was significantly decreased in comparison to control (*P* < 0.05). In comparison to the model group, the viability in the groups administered carbamazepine and high-, medium-, and low-dose JWCHSGD serum was significantly higher (*P* < 0.05) (Fig. 4).

### Flow cytometry detection of HT22 cell apoptosis

**Fig. 5:**
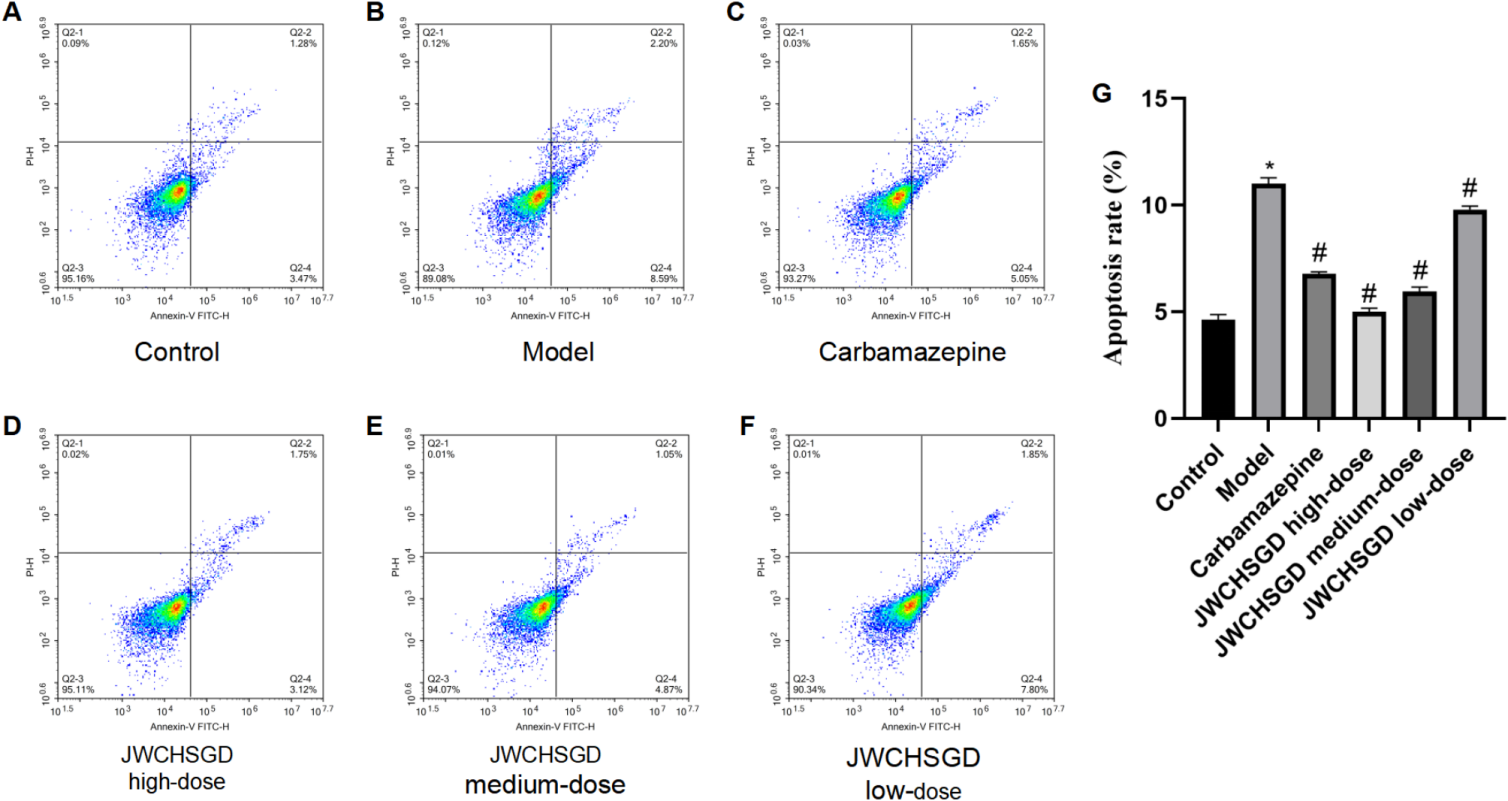
HT22 cell apoptosis. (**A–F**) Flow cytometry plots of HT22 cell apoptosis. (**G**) Bar graph of apoptosis rates. (*P < 0.05) model vs. control; (^#^*P* < 0.05) treatment vs. model.

Flow cytometry results showed that the percentage of apoptotic HT22 cells in the model group was significantly higher than in the control (*P* < 0.05). Treatment with carbamazepine and high-, medium-, and low-dose JWCHSGD serum resulted in different degrees of reduction in apoptosis (*P* < 0.05) (Fig. 5).

### Effect of JWCHSGD on relative mRNA expression of Circ_Csnk1g3, Csnk1g3-85aa, RIP1, RIP3, MLKL, TNF-α, IL-6, IL-1β in HT22 cells

**Fig. 6:**
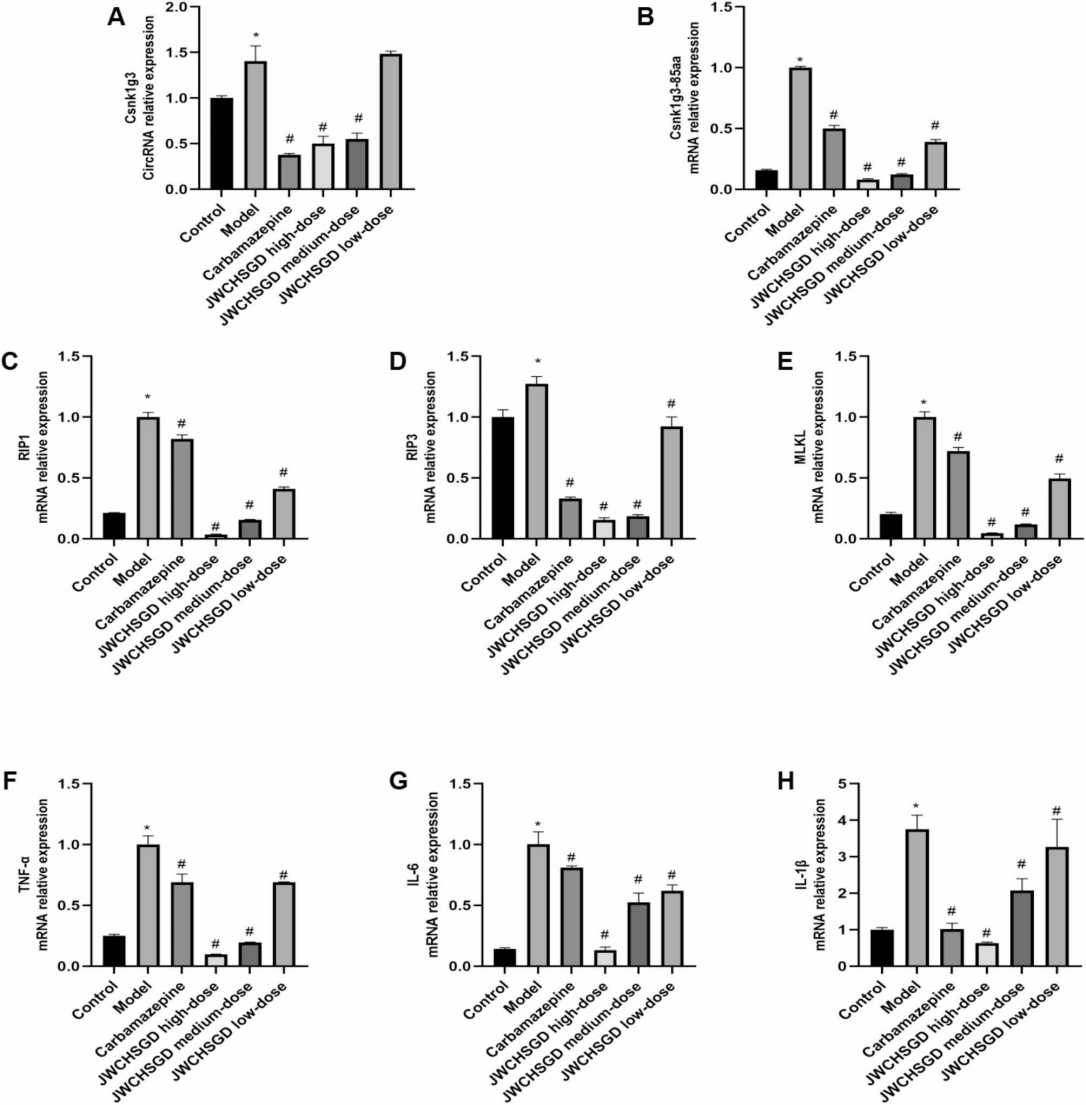
Relative mRNA expression of target genes in HT22 cells. (*P < 0.05) model vs. control; (^#^*P* < 0.05) treatments vs. model.

The PCR results showed that, compared with control, the mRNA expressions of *Circ_Csnk1g3*,* Csnk1g3-85aa*,* RIP1*,* RIP3*,* MLKL*,* TNF-α*,* IL-6*, and *IL-1β* were significantly higher in HT22 cells of the model group (*P* < 0.05). Compared with the model group, treatment with carbamazepine and high-, medium-, and low-dose JWCHSGD serum significantly reduced the mRNA expressions of these markers in HT22 cells (*P* < 0.05), with the greatest reduction observed in the carbamazepine group and the high-dose JWCHSGD group. Compared to the model group, the low-dose JWCHSGD group did not show a significant regulatory effect on Circ_Csnk1g3. This indicates that JWCHSGD (especially the high-dose JWCHSGD group) can inhibit expression of Circ_Csnk1g3, Csnk1g3-85aa, RIP1, RIP3, MLKL, TNF-α, IL-6, and IL-1β in HT22 epileptic cells (Fig. 6).

### Effect of JWCHSGD on relative protein expression of Csnk1g3-85aa, CK1γ3, RIP1, RIP3, MLKL, TNF-α, IL-6, and IL-1β in HT22 cells

**Fig. 7:**
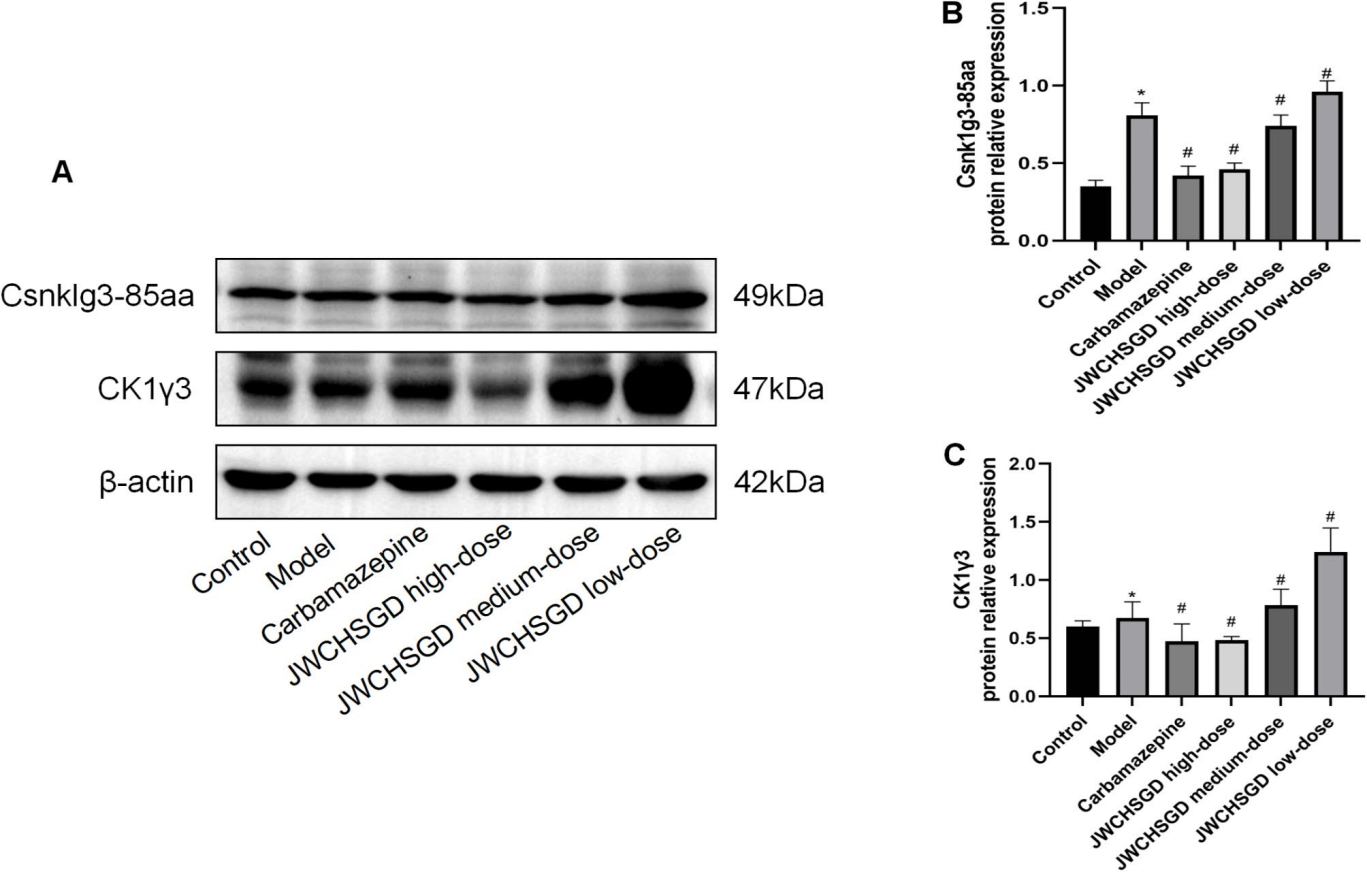
WB analysis of relative protein levels of target genes in HT22 Cells. (**A**) Electrophoresis of Csnk1g3-85aa, CK1γ3 protein expression in HT22 cells in each group; (**B**) relative protein level of Csnk1g3-85aa; (**C**) relative protein level of CK1γ3. (*P < 0.05) model vs. control; (^#^*P* < 0.05) treatment vs. model.

**Fig. 8:**
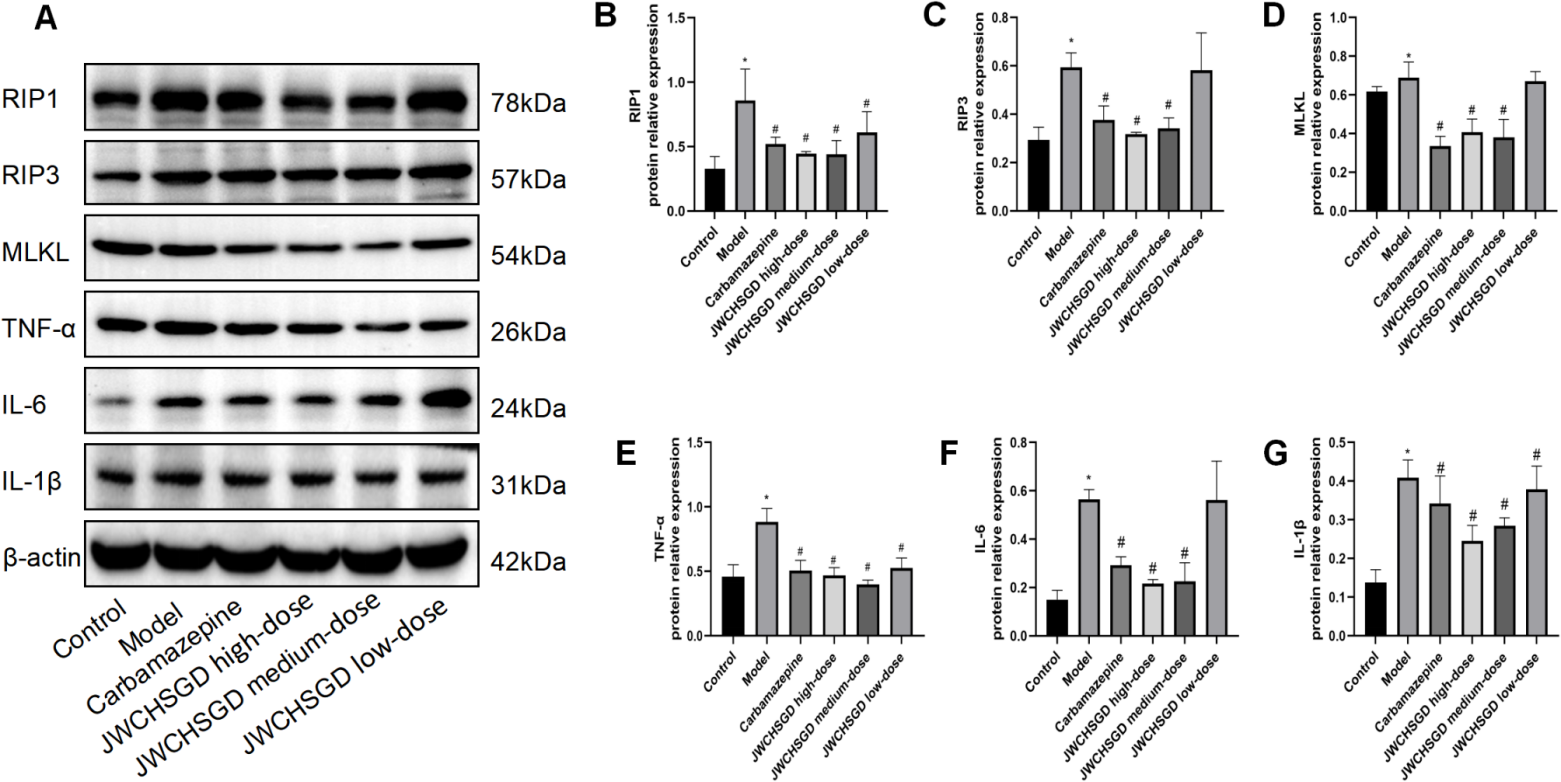
WB analysis of relative protein levels of target genes in HT22 Cells. (**A**) Electrophoresis of RIP1, RIP3, MLKL, TNF-α, IL-6, IL-1β protein expression in HT22 cells in each group. (**B**) Relative protein expression of RIP1, (**C**) RIP3, (**D**) MLKL, (**E**) TNF-α, (**F**) IL-6, (**G**) and IL-1β. (*P < 0.05) model vs. control; (^#^*P* < 0.05) treatment vs. model.

Compared to control, the protein expression of Csnk1g3-85aa, CK1γ3, RIP1, RIP3, MLKL, TNF-α, IL-6, and IL-1β was significantly elevated in HT22 cells of the model group (*P* < 0.05). In comparison with the model group, treatment with carbamazepine and high-, medium-dose JWCHSGD serum significantly reduced the protein expressions of these markers in HT22 cells (*P* < 0.05), with the greatest reduction observed in the carbamazepine group and the high-dose JWCHSGD group. Compared to the model group, the low-dose JWCHSGD group slightly increased the protein expression levels of Csnk1g3-85aa, CK1γ3, RIP1, TNF-α, and IL-1β (*P* < 0.05). The results show that JWCHSGD can inhibit the protein expression of Csnk1g3-85aa, CK1γ3, RIP1, RIP3, MLKL, TNF-α, IL-6, and IL-1β in HT22 epileptic cells, exhibiting a dose-dependent pattern (Figs. 7 and 8).

### Localization of injected adenovirus

**Fig. 9:**
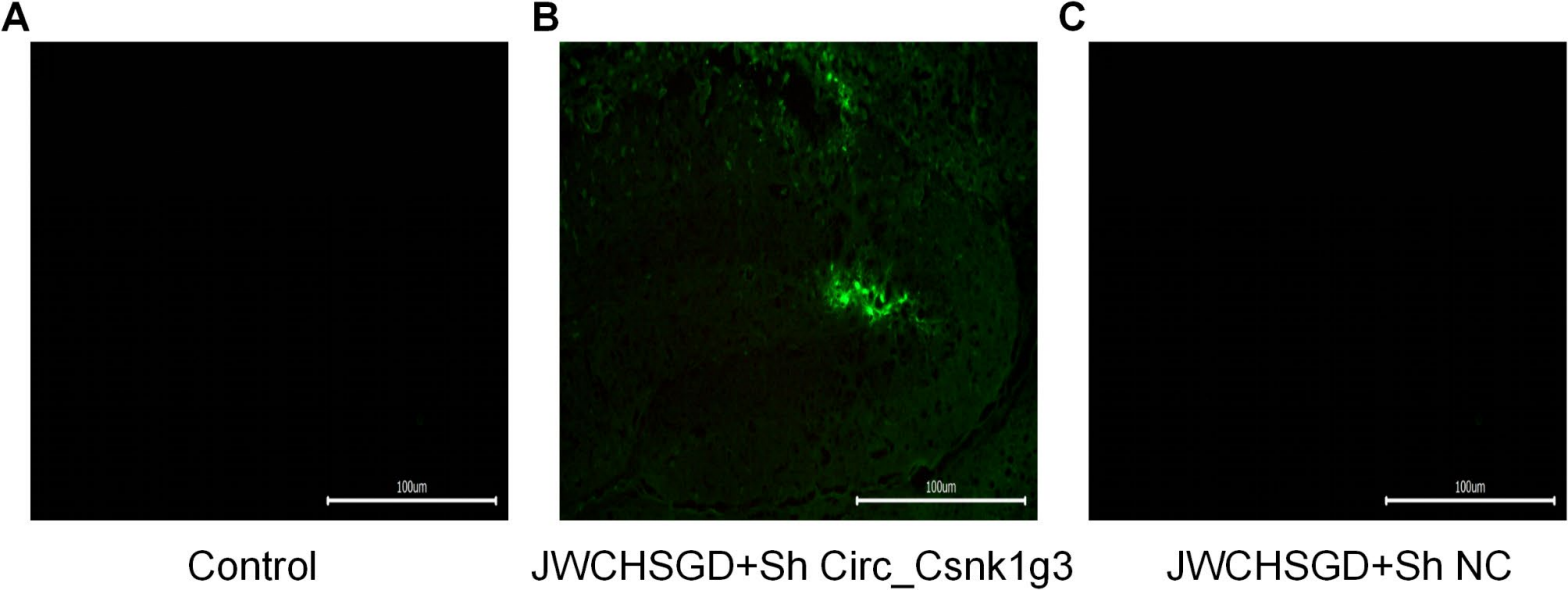
Localization of FITC-tagged interfering adenovirus (green) in frozen sections (excitation at 465–495 nm, emission at 515–555 nm). (**A**) Control group; (**B**) JWCHSGD + Sh Circ_Csnk1g3 group; (**C**) JWCHSGD + Sh NC group.

Brain tissue sections from control, JWCHSGD + Sh Circ_Csnk1g3, and JWCHSGD + Sh NC groups were placed on sample holders with the tissue side up. OCT embedding medium was applied around the tissue, and the sample holders were placed on the freezing platform of a cryostat for rapid freezing and embedding. Once the OCT turned white and hardened, sections were cut, examined under a fluorescence microscope and images were obtained. The slices from the JWCHSGD + Sh Circ_Csnk1g3 group exhibited green fluorescence, while control and JWCHSGD + Sh NC groups showed no fluorescence, indicating successful localization of the Circ_Csnk1g3 interfering adenovirus in mice (Fig. 9).

### EEG changes in mice

**Fig. 10:**
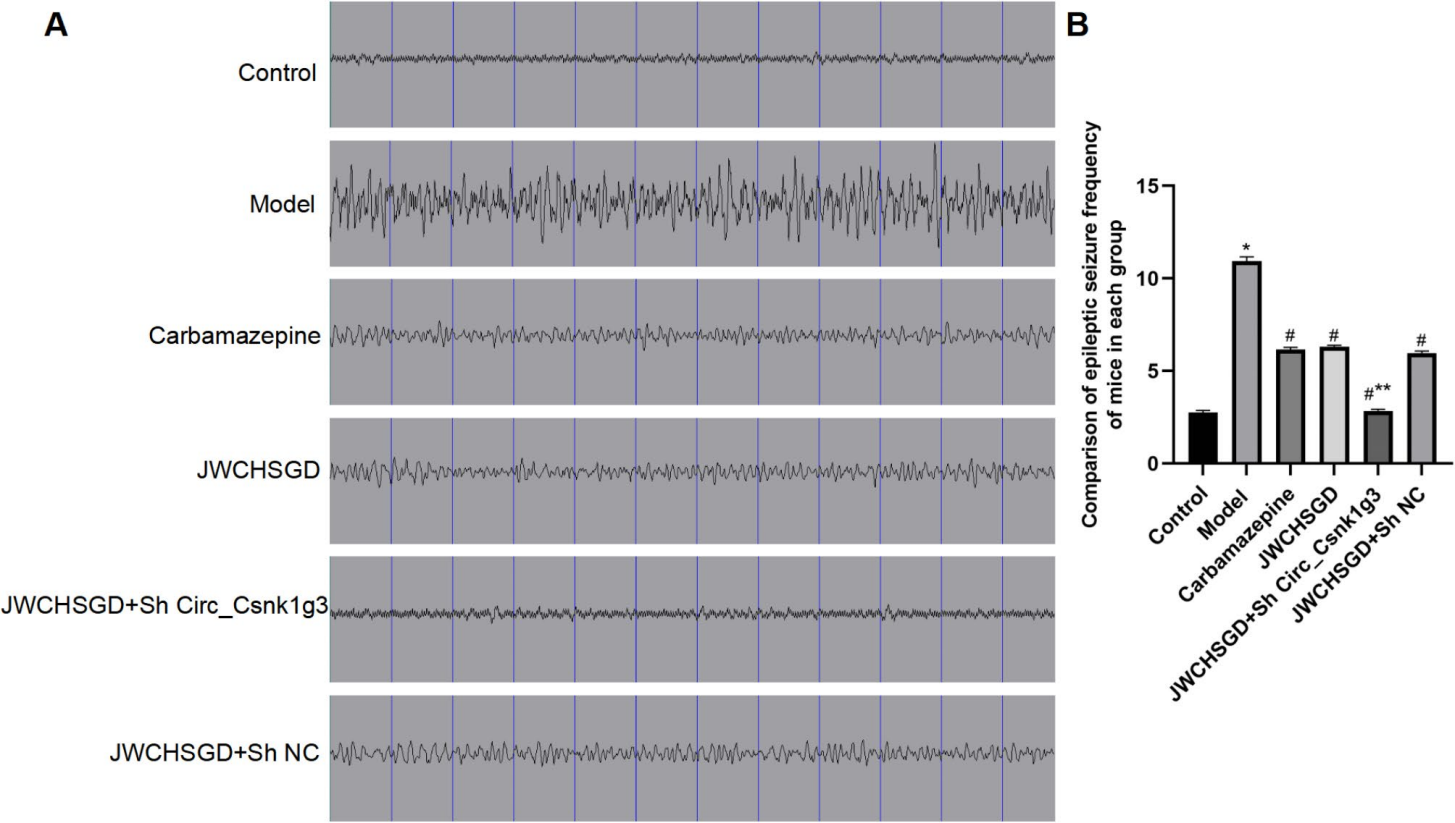
Electroencephalograms of mice in each group (*n* = 10, repeated three times). (**A**) EEGs; (**B**) statistical comparisons of frequency of epileptic seizures for each group. (*P < 0.05) model vs. control; (^#^*P* < 0.05) treatments vs. model; (**P < 0.05) JWCHSGD + Sh Circ_Csnk1g3 vs. JWCHSGD.

The EEG results showed that compared to control, the model mice exhibited continuous high-frequency, high-amplitude, multi-spike epileptiform discharges. In comparison with the model group, the frequencies and intensities of epileptiform discharges were significantly reduced in the carbamazepine group, JWCHSGD group, JWCHSGD + Sh Circ_Csnk1g3 group, and JWCHSGD + Sh NC group, with the JWCHSGD + Sh Circ_Csnk1g3 group showing the greatest significant reduction, followed by the carbamazepine, JWCHSGD, and JWCHSGD + Sh NC group (Fig. 10).

### Effect of JWCHSGD on hippocampal tissues from epileptic mice

**Fig. 11:**
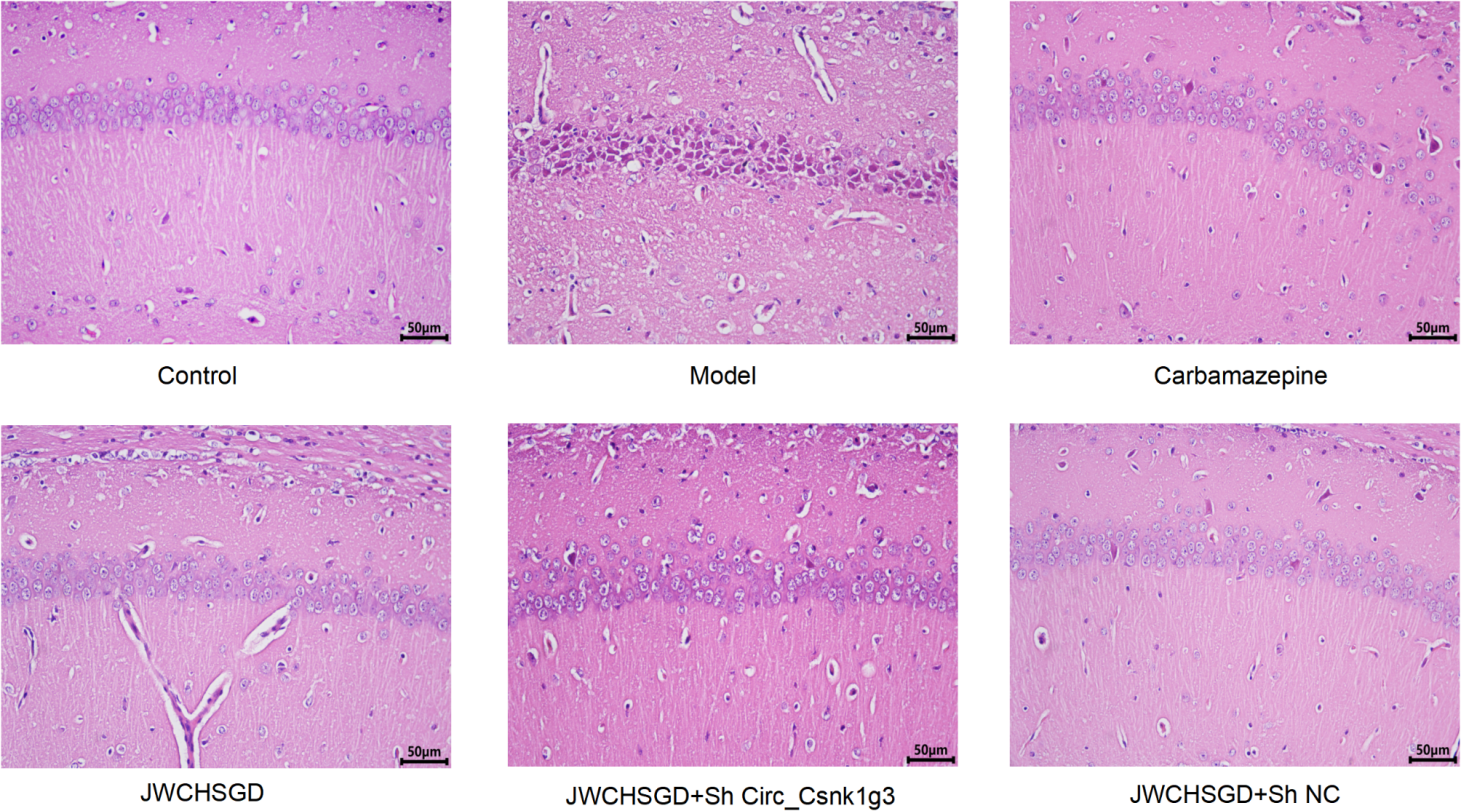
H&E staining of hippocampal tissues from each group (×400).

H & E staining results observed with a visible-light microscope indicated that the hippocampal neurons in the control sections were well-organized, with clear layers, intact structure, full nuclei, and no significant pathological changes. In the model group, hippocampal tissue showed obvious damage, with neurons appearing polygonal, nuclei condensed and deeply stained, and extensive neuronal necrosis. In the carbamazepine group, JWCHSGD group, JWCHSGD + Sh Circ_Csnk1g3 group, and JWCHSGD + Sh NC group, hippocampal neurons were well-organized, with clear layers, intact structure, full nuclei, and only occasional polygonal neuronal cell bodies and condensed nuclei, indicating a marked improvement in pathological damage in comparison with the model group, with the JWCHSGD + Sh Circ_Csnk1g3 group showing the most noticeable therapeutic effect (Fig. 11).

### Effect of JWCHSGD on apoptosis of hippocampal neurons in epileptic mice

**Fig. 12:**
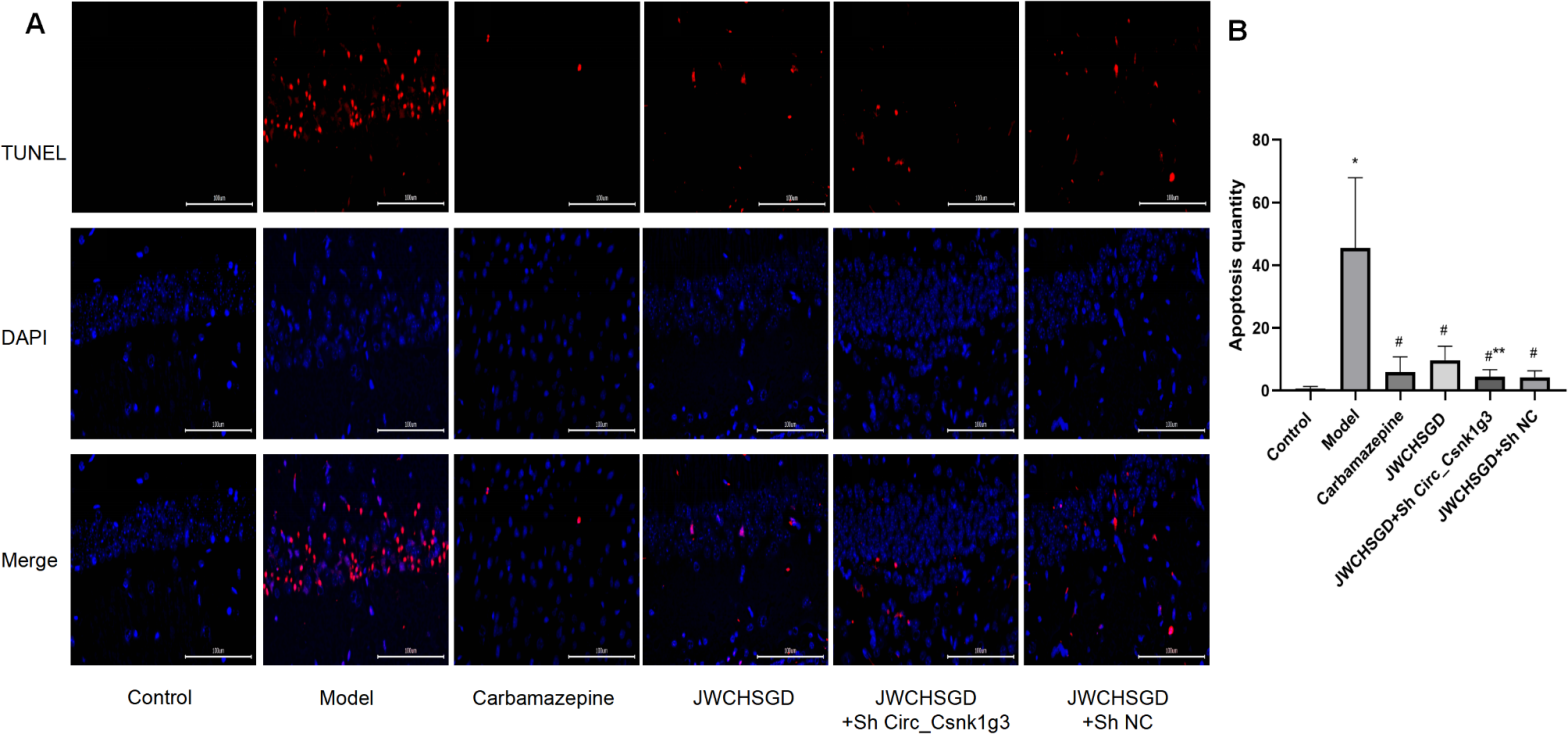
TUNEL staining to determine hippocampal apoptosis (*n* = 10, repeated three times). (**A**) Images of TUNEL staining (red) in each group (×400). (**B**) Statistical comparison of TUNEL-stained tissues. (*P < 0.05) model vs. control; (^#^*P* < 0.05) treatments vs. model; (**P < 0.05) JWCHSGD + Sh Circ_Csnk1g3 vs. JWCHSGD.

In comparison with the control, the model mice exhibited significantly more TUNEL-positive cells in hippocampal tissues (*P* < 0.05). Compared with the model group, the number of TUNEL-positive cells in the hippocampus was significantly reduced in the carbamazepine, JWCHSGD, JWCHSGD + Sh Circ_Csnk1g3, and JWCHSGD + Sh NC groups (*P* < 0.05). The JWCHSGD + Sh Circ_Csnk1g3 group demonstrated the most effective inhibition of apoptosis (Fig. 12).

### Effect of JWCHSGD on relative mRNA expression of Circ_Csnk1g3, Csnk1g3-85aa, RIP1, RIP3, MLKL, TNF-α, IL-6, and IL-1β in hippocampal tissues of epileptic mice

**Fig. 13:**
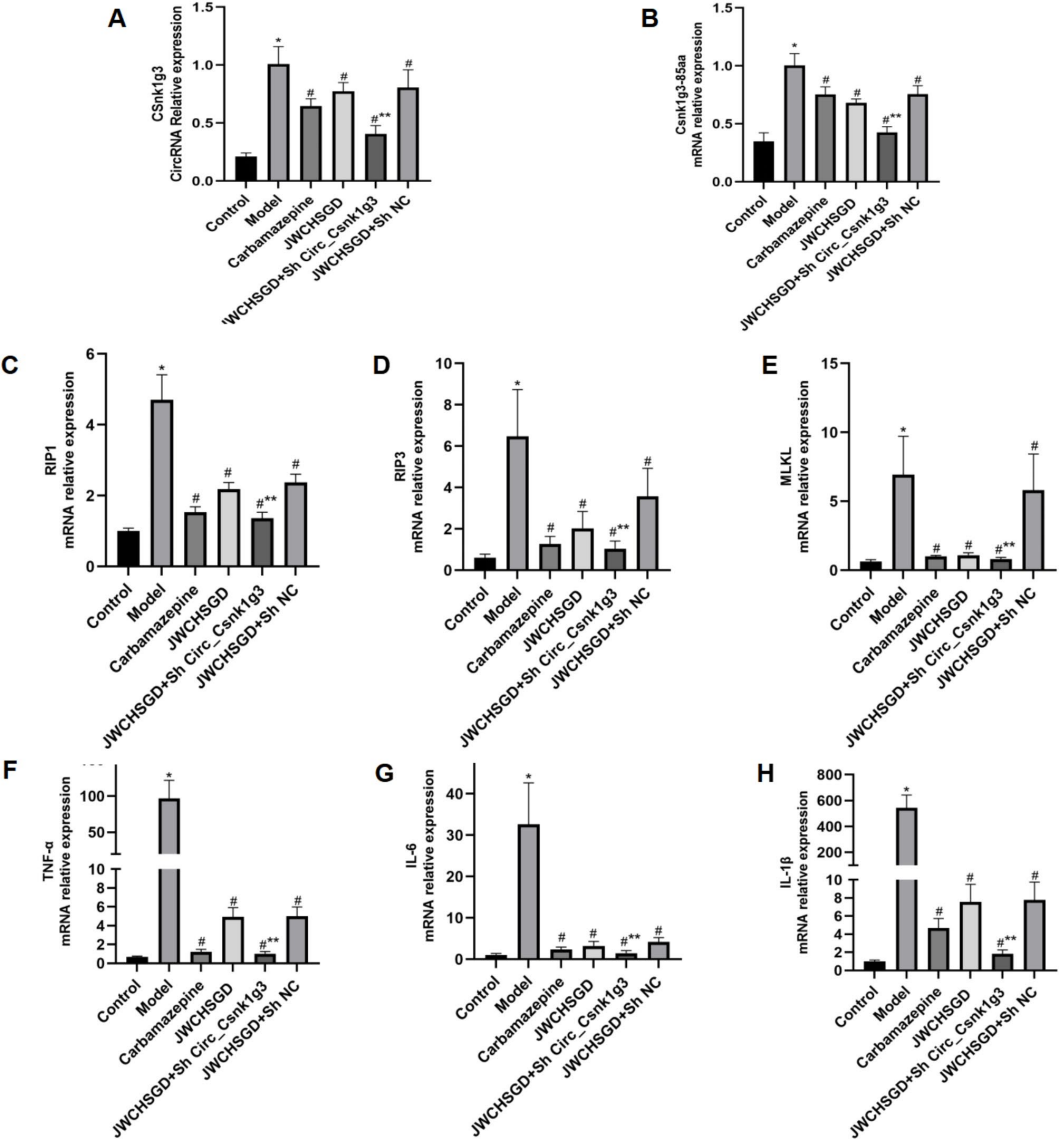
PCR analysis of relative mRNA expression of target genes in hippocampal tissues from mice in each group (*n* = 10, repeated three times). (*P < 0.05) model vs. control; (^#^*P* < 0.05) treatments vs. model; (**P < 0.05) JWCHSGD + Sh Circ_Csnk1g3 vs. JWCHSGD.

In comparison with the control, the model group showed significantly increased mRNA expression of Circ_Csnk1g3, Csnk1g3-85aa, RIP1, RIP3, MLKL, TNF-α, IL-6, and IL-1β in hippocampal tissue (*P* < 0.05). In comparison to the model group, the carbamazepine, JWCHSGD, JWCHSGD + Sh Circ_Csnk1g3, and JWCHSGD + Sh NC groups showed significantly reduced mRNA expression of these genes (*P* < 0.05), with the JWCHSGD + Sh Circ_Csnk1g3 group showing the largest reduction. This shows that JWCHSGD can inhibit the mRNA expression of Circ_Csnk1g3, Csnk1g3-85aa, RIP1, RIP3, MLKL, TNF-α, IL-6, and IL-1β in hippocampal tissue from epileptic mice (Fig. 13).

### Effect of JWCHSGD on relative protein expression of Csnk1g3-85aa, CK1γ3, RIP1, RIP3, MLKL, TNF-α, IL-6, and IL-1β in hippocampal tissues of epileptic mice

**Fig. 14:**
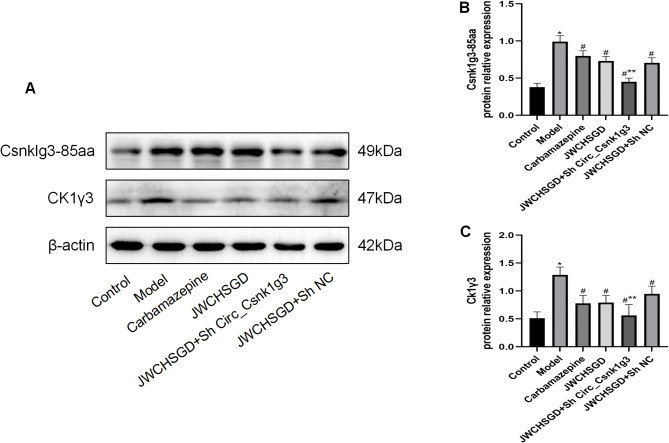
WB analysis of relative protein expression of target genes in hippocampal tissues of mice (*n* = 10, repeated three times). (**A**) Detection of Csnk1g3-85aa and CK1γ3 protein expression in each group. (**B**) Csnk1g3-85aa. (**C**) CK1γ3. (*P < 0.05) model vs. control; (^#^*P* < 0.05) treatments vs. model; (**P < 0.05) JWCHSGD + Sh Circ_Csnk1g3 vs. JWCHSGD.

**Fig. 15:**
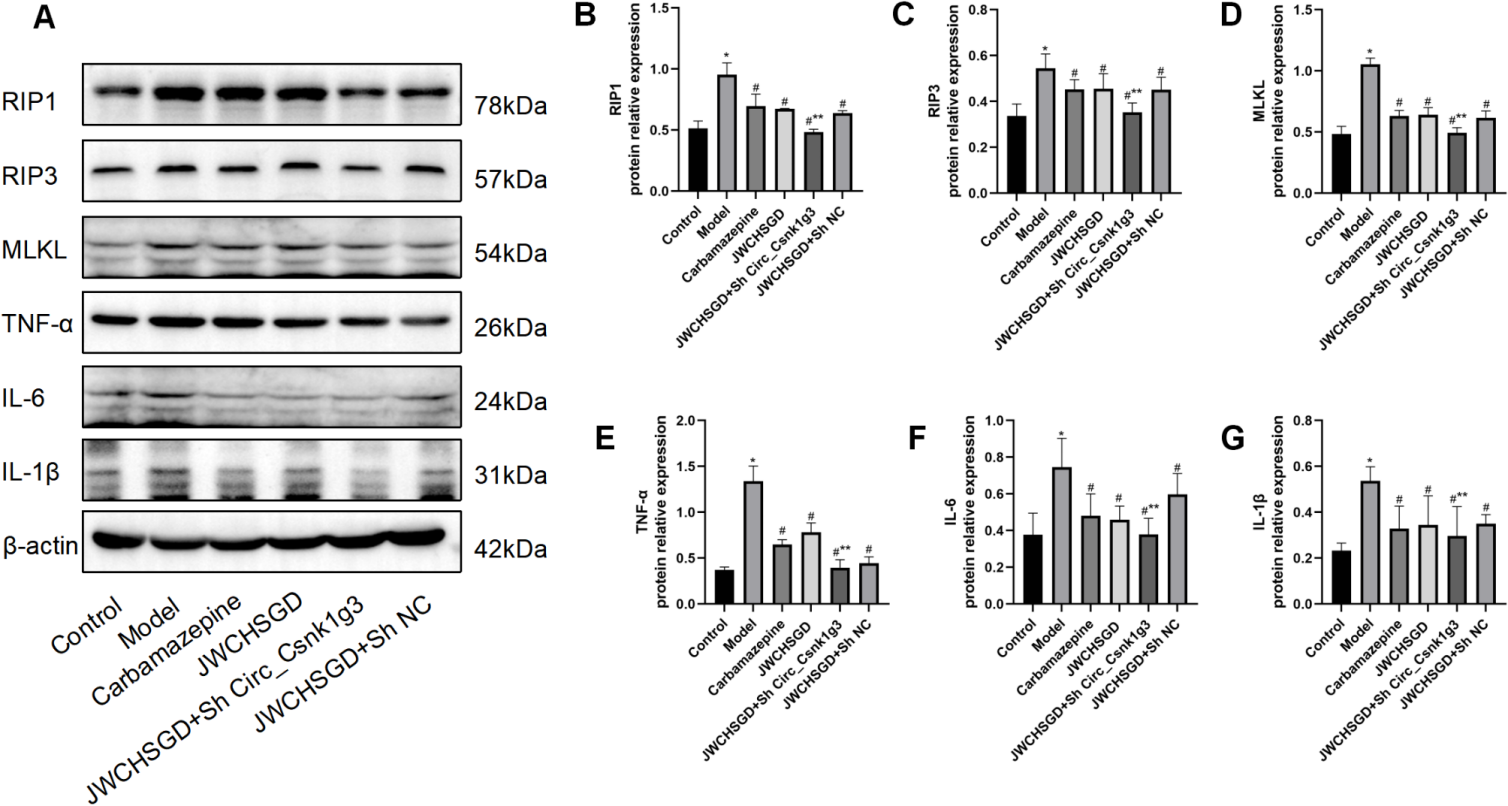
WB analysis of relative protein expression of target genes in hippocampal tissues of mice (*n* = 10, repeated three times). (**A**) Detection of RIP1, RIP3, MLKL, TNF-α, IL-6, IL-1β protein expression in each group. (**B**) RIP1; (**C**) RIP3; (**D**) MLKL; (**E**) TNF-α; (**F**) IL-6; (**G**) IL-1β. (*P < 0.05: model vs. control, (^#^*P* < 0.05) treatments vs. model; (**P < 0.05) JWCHSGD + Sh Circ_Csnk1g3 vs. JWCHSGD.

Compared with control, the model group had significantly higher protein expression of Csnk1g3-85aa, CK1γ3, RIP1, RIP3, MLKL, TNF-α, IL-6, and IL-1β in hippocampal tissue (*P* < 0.05). In comparison to the model group, the carbamazepine, JWCHSGD, JWCHSGD + Sh Circ_Csnk1g3 group, and JWCHSGD + Sh NC groups exhibited significantly decreased expression of these markers (*P* < 0.05), with the JWCHSGD + Sh Circ_Csnk1g3 group showing the greatest reduction. This supports our hypothesis that JWCHSGD inhibits the protein expression of Csnk1g3-85aa, CK1γ3, RIP1, RIP3, MLKL, TNF-α, IL-6, and IL-1β in hippocampal tissues of epileptic mice (Figs. 14 and 15).

## Discussion

Recent studies have shown that circRNA in non-coding RNAs can participate in the development of epilepsy by regulating inflammation, neuronal excitability, synaptic plasticity, and neurotransmitter release^25^. CircRNA is abundantly expressed in the nervous system^26^ and increasing evidence has shown that circRNA can associate with RNA-binding proteins (RBPs) to exert biological functions and influence epileptic seizures^27,28^. Additionally, there is evidence suggesting that circRNA can code for certain proteins with potential biological functions^29^.

Investigations of the process of circRNA translation mainly focused on non-coding regions of the genome, which contain many small open reading frames (sORFs). The translation products of these sORFs are known as small ORF-encoded peptides (SEPs) or micro-peptides. Some micro-peptides have been confirmed to be stably present within cells and play important roles independent of the RNA sources^30–36^. CircRNA, with its circular structure and partial exonic sequences of certain genes, contains potential ORFs and has translation potential. Most circRNA mainly exists in the cytoplasm, which spatially facilitates its translation^37^. Studies have shown that circRNA contains multiple ORFs and can achieve ribosomal entry through a 5′ m7G cap-independent pathway, including translational initiation from an internal ribosome entry site (IRES) and alteration in m6A methylation patterns^38,39^. For instance, research has reported that through IRES1, circSTX6 encodes a novel 144-amino-acid peptide, circSTX6-144aa, which independently promotes the progression of hepatocellular carcinoma (HCC)^40^.

The JWCHSGD formulation contains Chaihu (Bupleurum) as the chief herb to regulate liver qi and relieve stagnation. Xiangfu (*Cyperus rotundus*) and Chuanxiong (Ligusticum chuanxiong) are used to soothe the liver, regulate qi, promote blood flow, and relieve pain. These two herbs complement each other and serve as assistant herbs in the formulation. Chenpi (*Citrus reticulata*) and Zhike (*Aurantii Fructus*) adjust qi movement, while Baishao (*Paeonia lactiflora*) nourishes the blood and softens the liver. Zhebeimu (*Fritillaria*) and Gouteng (*Uncaria*) both have effects on calming the liver, extinguishing wind, and stopping spasms. This combination effectively addresses liver qi stagnation, promotes qi movement, and resolves phlegm and wind. Modern pharmacological research indicates that the primary active components in JWCHSGD include triterpenoids, saponins, flavonoids, and phenolic acids, with key ingredients like kaempferol, β-sitosterol, isorhamnetin, quercetin, naringenin, fritillaria alkaloids, gouteng alkaloids, paeoniflorin, and myricetin. These compounds possess an array of pharmacological effects, such as anti-epileptic, anti-inflammatory, antioxidant, sedative, immune-regulating, and neuroprotective activities^18,41^. JWCHSGD is widely used in clinical settings to treat neurological disorders, including epilepsy^12,42–46^.

In this study, by constructing an HT22 epilepsy cell model, we found that treatment with carbamazepine and JWCHSGD. at different concentrations significantly increased HT22 viability and markedly reduced apoptosis as detected by flow cytometry. RT-PCR revealed a significant decrease in the relative expression level of Circ_Csnk1g3, Csnk1g3-85aa, RIP1, RIP3, MLKL, TNF-α, IL-6, and IL-1β mRNA. Protein immunoblotting also showed a notable reduction in the relative protein levels of Csnk1g3-85aa, CK1γ3, RIP1, RIP3, MLKL, TNF-α, IL-6, and IL-1β. Moreover, mice treated with high-dose JWCHSGD showed the most pronounced therapeutic effect. It is worth mentioning that in this experiment, SD rat blood was used for the cell experiments, while C57BL mice were used for in vivo behavioral assessments. This choice was mainly due to the need for a relatively large volume of drug-containing serum, making SD rats a practical choice to prepare serum for the cell experiments^47,48^. In terms of mechanisms, we constructed an epileptic mouse model and found that following treatment with carbamazepine and JWCHSGD, and knockdown of Circ_Csnk1g3, the frequency and intensity of epileptic discharges in the EEGs of the epileptic mice were significantly reduced. H&E staining showed improvements in the pathological structure and cell morphology of the hippocampus, with the JWCHSGD + Sh Circ_Csnk1g3 group showing the best therapeutic effect. However, Nissl staining is an important tool in neuroscience research as it highlights Nissl bodies in the neuronal cytoplasm, revealing detailed neuronal structures. Not using Nissl staining to observe hippocampal neurons is one of the limitations of this study. TUNEL staining revealed that the percentage of TUNEL-positive hippocampal cells in the epileptic mice was significantly reduced, with the JWCHSGD + Sh Circ_Csnk1g3 group showing the most effective suppression of neuronal apoptosis. Real-time PCR and protein immunoblotting further confirmed a significant reduction in the relative expression levels of Circ_Csnk1g3, Csnk1g3-85aa, RIP1, RIP3, MLKL, TNF-α, IL-6, and IL-1β mRNA and proteins, with the JWCHSGD + Sh Circ_Csnk1g3 group showing the best results. This showed that the anti-epileptic effect of JWCHSGD was dose-dependent and that it could act through down-regulating circRNA-Csnk1g3 expression in hippocampal neurons, thereby activating the anti-epileptic process associated with the Csnk1g3-85aa/ CK1γ3/TNF-α signaling pathway.

In this experiment, JWCHSGD inhibited the translation of circRNA-Csnk1g3 into polypeptides, which affected necroptosis in hippocampal neurons and thereby reduced the occurrence of epilepsy. This finding aligns with the mechanism proposed by Jin et al.^49^. Specifically, JWCHSGD regulates the translation of circRNA-Csnk1g3 into proteins, impacting the downstream classical necroptosis signaling pathway and significantly suppressing the gene expression of proteins such as RIPK1, RIPK3, and MLKL. Under stress conditions, when the RIPK1/RIPK3/MLKL pathway is activated, MLKL oligomerizes, and these oligomers bind to phosphatidylinositol lipids and cardiolipin, causing MLKL to translocate from the cytoplasm to the cell membrane. This directly disrupts membrane integrity and triggers necroptosis^50,51^. TNF-α, neogenin, and calpain/JNK1 pathways are involved in initiating necroptosis signaling and are closely linked to the RIP1/RIP3/MLKL pathway^52^. Among these, the TNF-α pathway increases the levels of pro-inflammatory cytokines TNF-α, IL-1β, and IL-6, disrupts the blood-brain barrier, and participates in hsp90-mediated necrosome formation and necroptosis, ATP depletion, and calcium imbalance, which collectively trigger and exacerbate seizures^53–57^. The experimental results demonstrate that JWCHSGD can significantly reverse these phenomena, including inhibiting the expression of circRNA-Csnk1g3 and its translated polypeptides, thereby suppressing the expression of RIP1/RIP3/MLKL signaling proteins and ultimately improving the biological effects of hippocampal neuronal necroptosis. The role of necroptosis in the mechanism of epilepsy is a crucial direction for future basic and clinical research in developing targeted antiepileptic drugs. Our findings suggest that JWCHSGD has therapeutic potential for epilepsy by inhibiting hippocampal neuronal necroptosis, providing direction and data support for future clinical translation and in-depth research and development.

In this study, we validated that Circ_Csnk1g3 encoded a novel peptide, Csnk1g3-85aa, which was significantly increased in epileptic hippocampal tissue and an HT22 cell model of epilepsy. JWCHSGD. inhibited the expression of Csnk1g3-85aa in both epileptic hippocampal tissue and HT22 cells. Our findings provide several new insights. Circ_Csnk1g3 may serve as a diagnostic marker for epilepsy, improving diagnostic accuracy and sensitivity. Circ_Csnk1g3 participates in necroptosis and inflammatory signaling pathways in epilepsy by regulating the encoding peptide Csnk1g3-85aa, showing significant potential as a new therapeutic target and diagnostic marker for epilepsy. However, due to the numerous variables in the study, further research is needed to determine whether Circ_Csnk1g3 specifically regulates Csnk1g3-85aa to inhibit necroptosis and inflammatory responses, and whether the association between the peptide Csnk1g3-85aa and downstream signaling pathways is specific. This study also revealed a novel molecular mechanism of JWCHSGD. in the prevention and treatment of epilepsy, as manifested in its regulation of the Circ_Csnk1g3/Csnk1g3-85aa/ CK1γ3/TNF-α pathway to inhibit hippocampal neuronal necroptosis and inflammation.

## Electronic supplementary material

Below is the link to the electronic supplementary material.

Supplementary Material 1

## Data Availability

Data are available from the corresponding author on reasonable request.
