# Supplementary material for: Mitigating effects of Jiawei Chaihu Shugan decoction on necroptosis and inflammation of hippocampal neurons in epileptic mice
Source: Sci Rep. 2025 Feb 7;15:4649. doi: 10.1038/s41598-025-89275-8 (PMC11805973; doi:10.1038/s41598-025-89275-8)
Supplement: Supplementary file 1 — Supplementary Material 1 [file 41598_2025_89275_MOESM1_ESM.docx]

**Supplementary Information**

Original images of Western Blot experiment in this study:

**1. Original Western Blot images *in vitro***


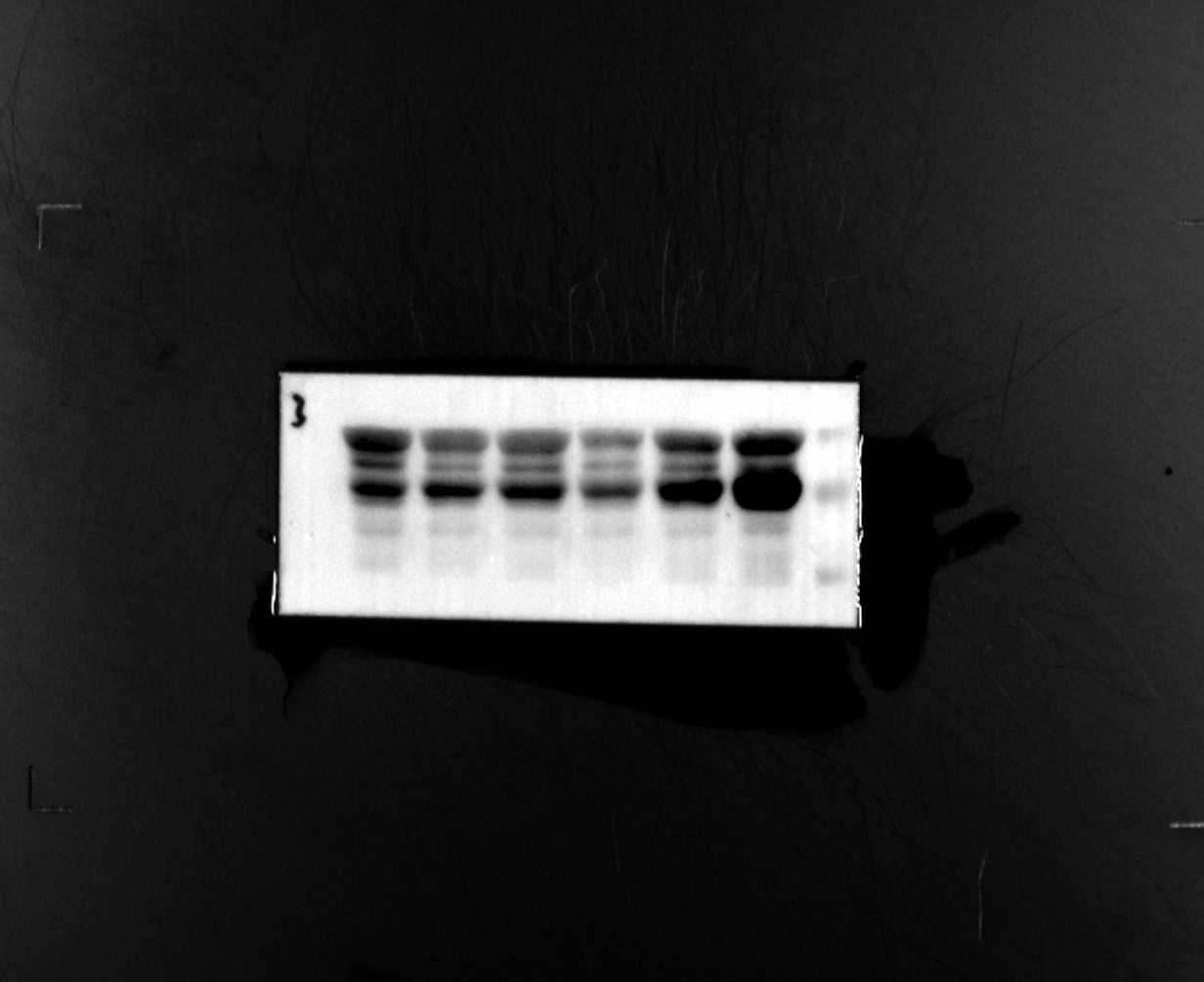

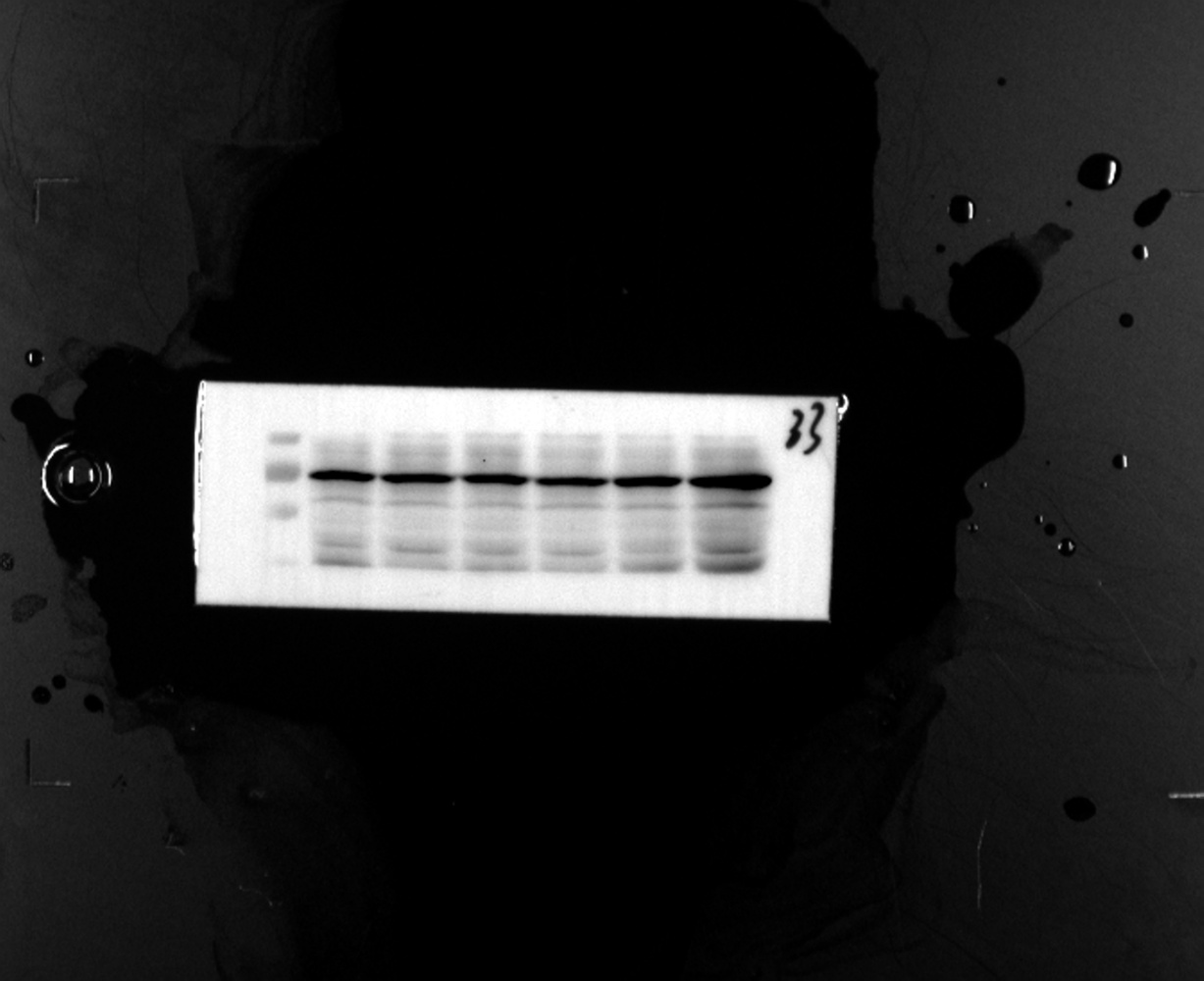


CK1γ3 Csnk1g3-85aa


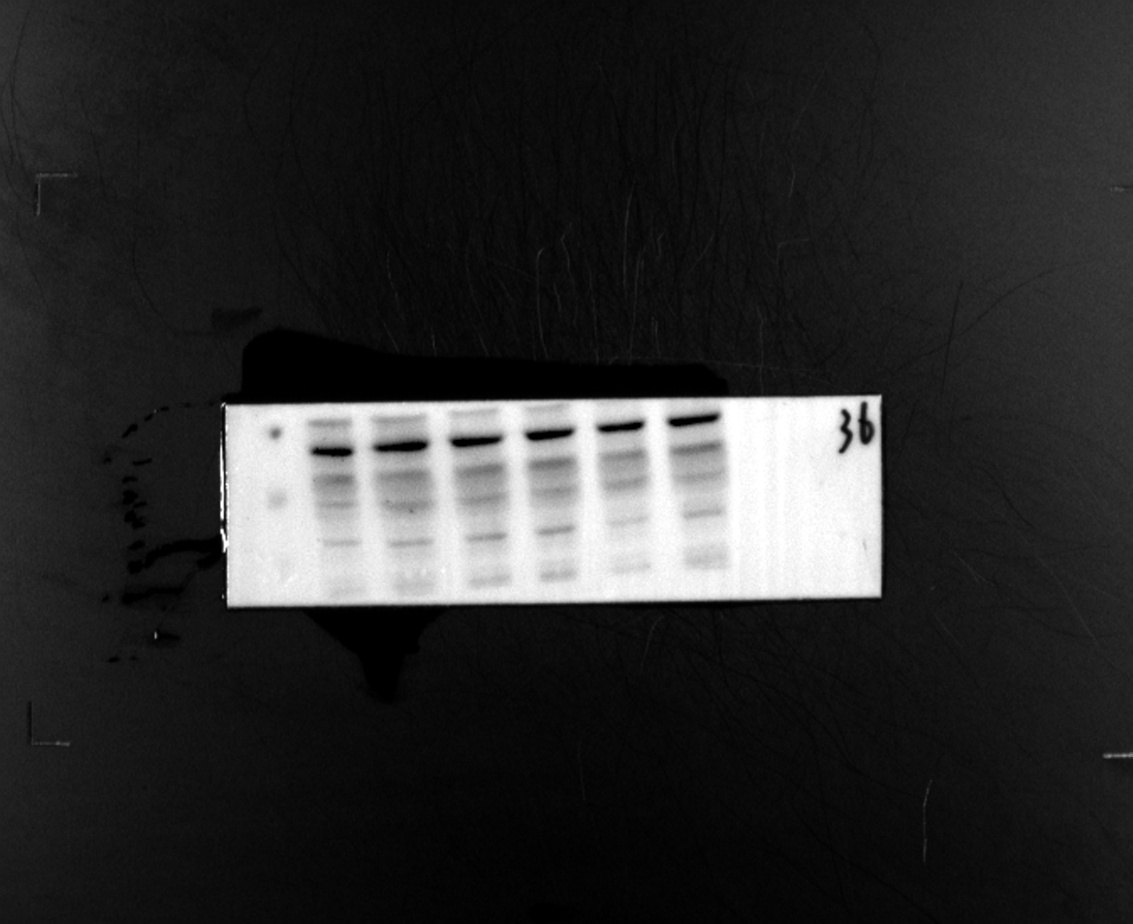

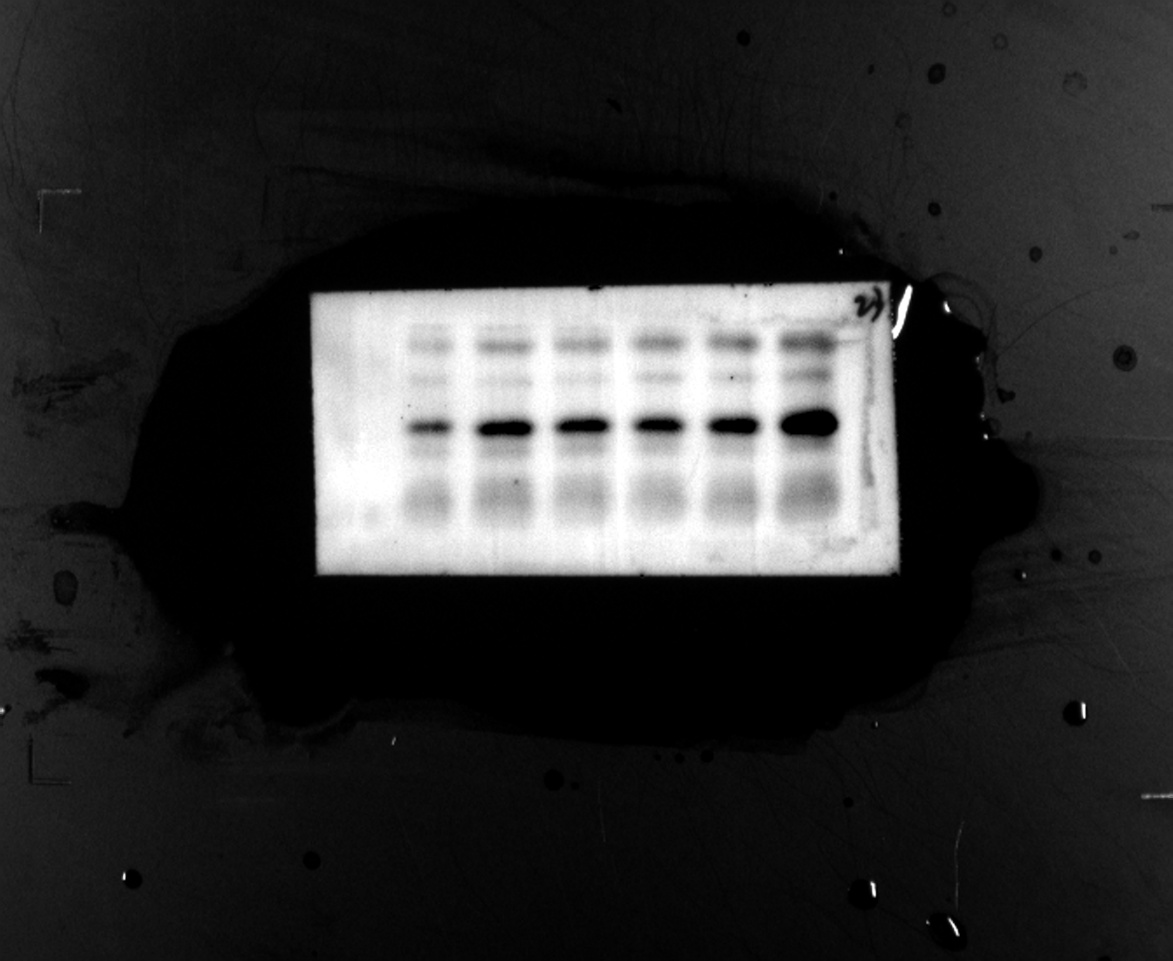


IL-1β IL-6


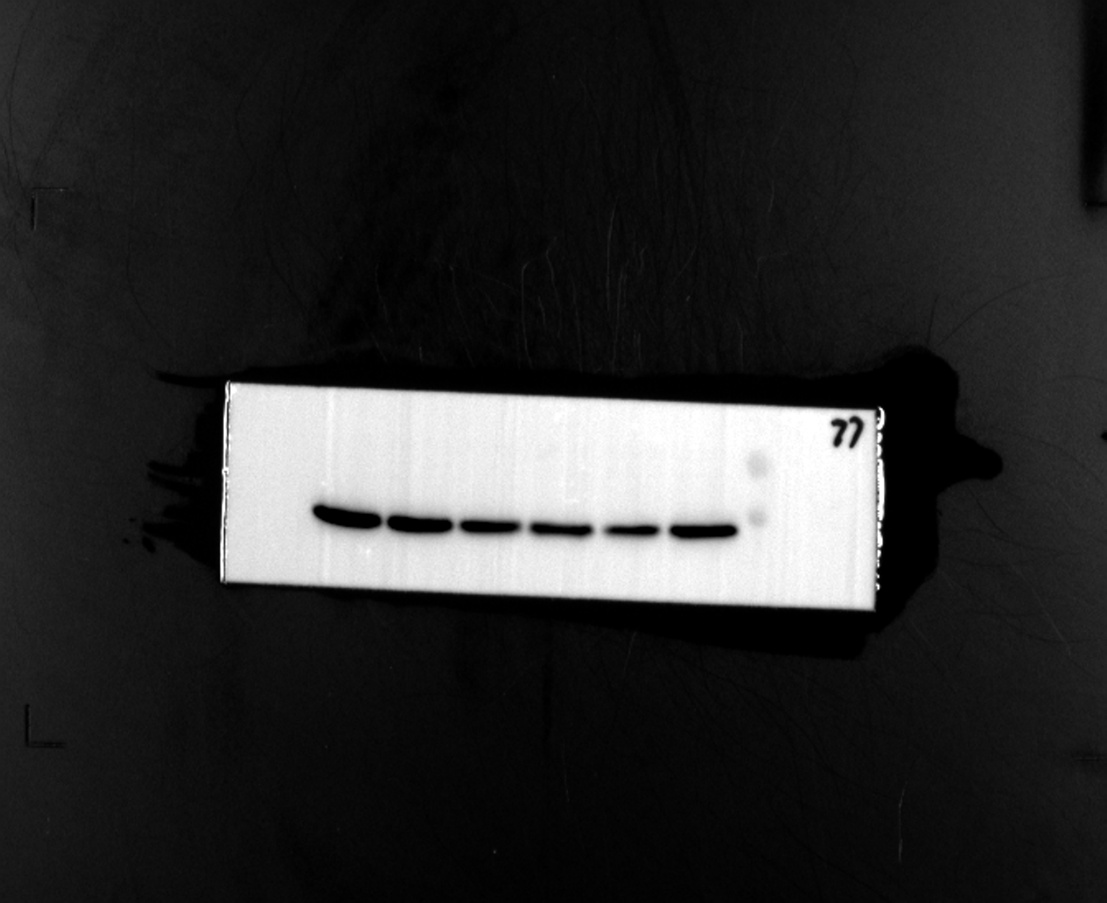

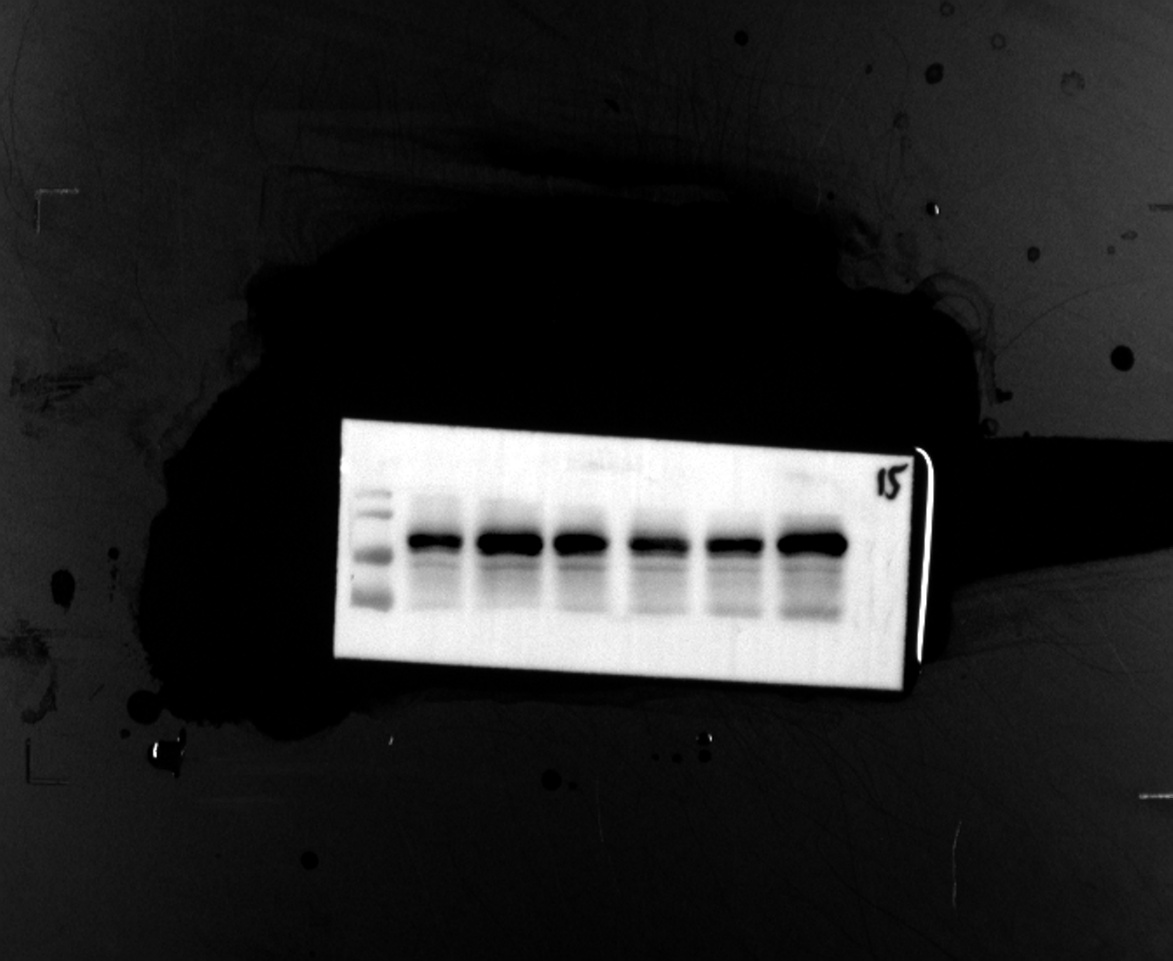


MLKL RIP1


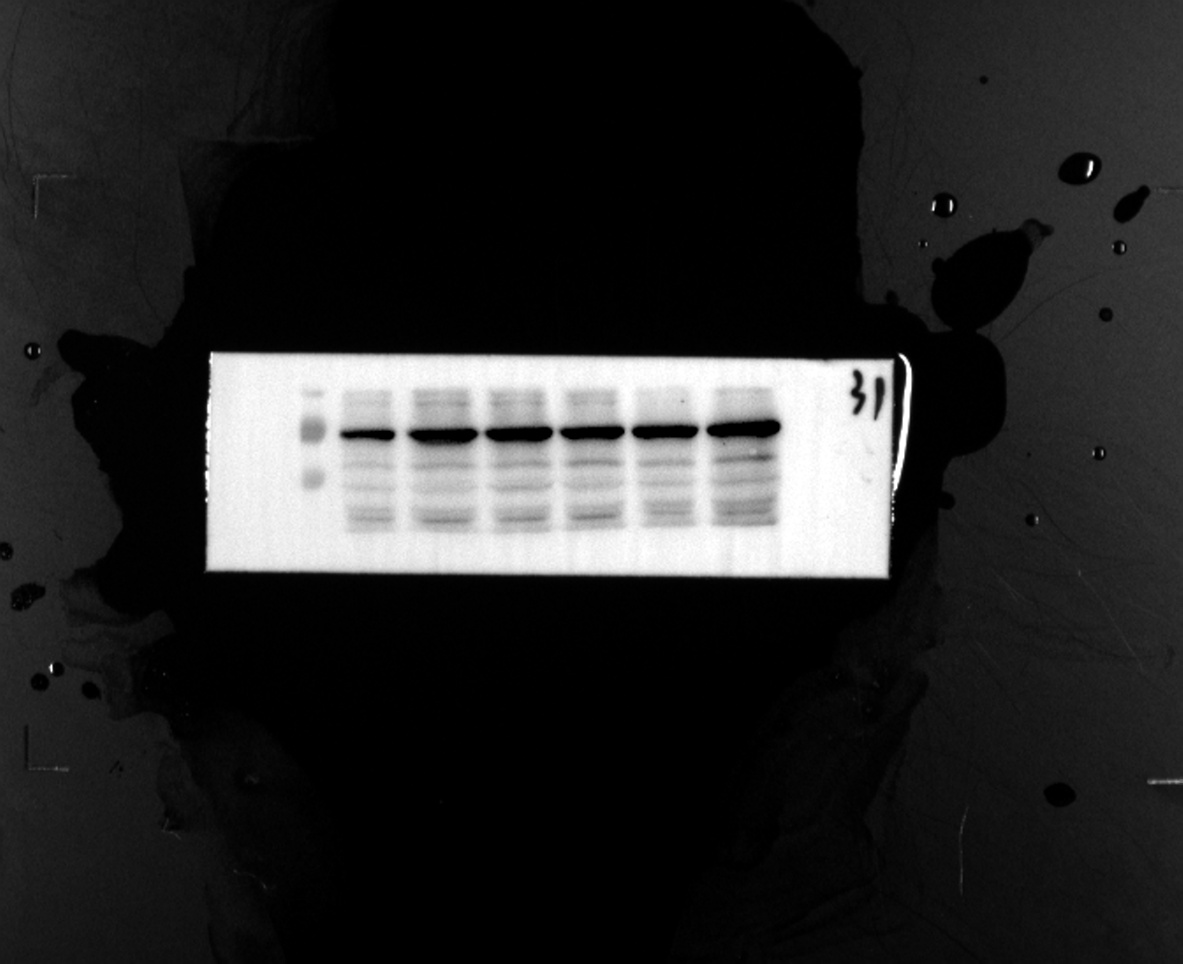

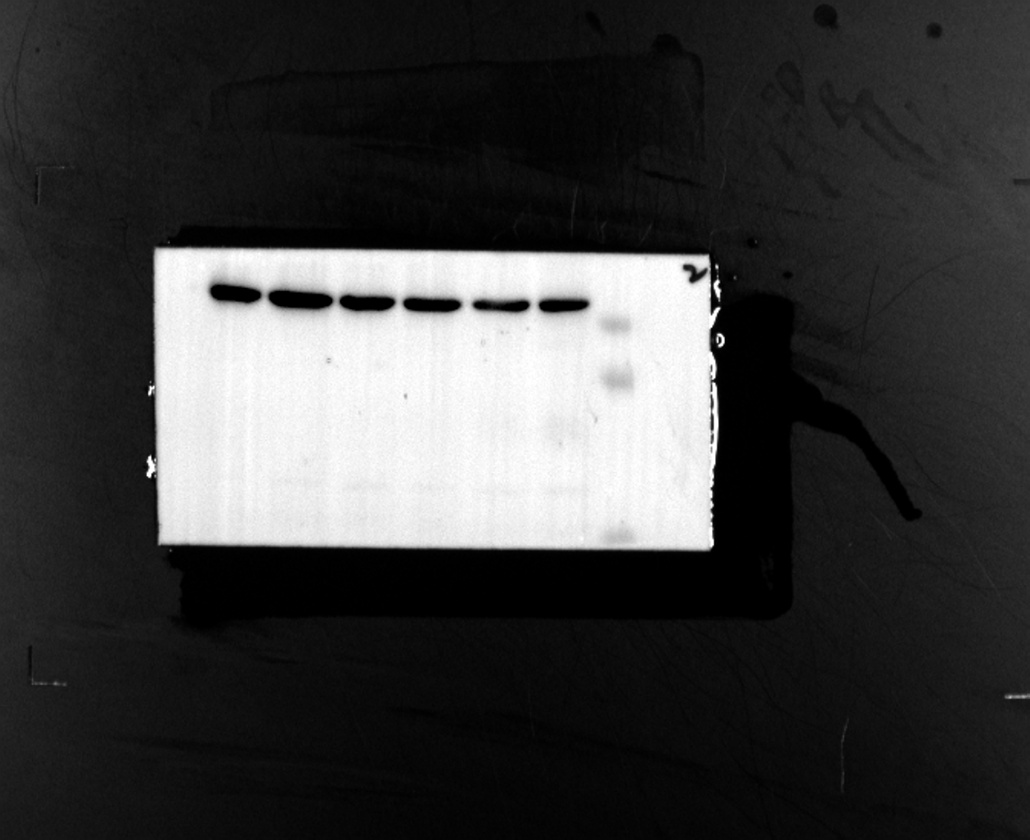


RIP3 TNF-α


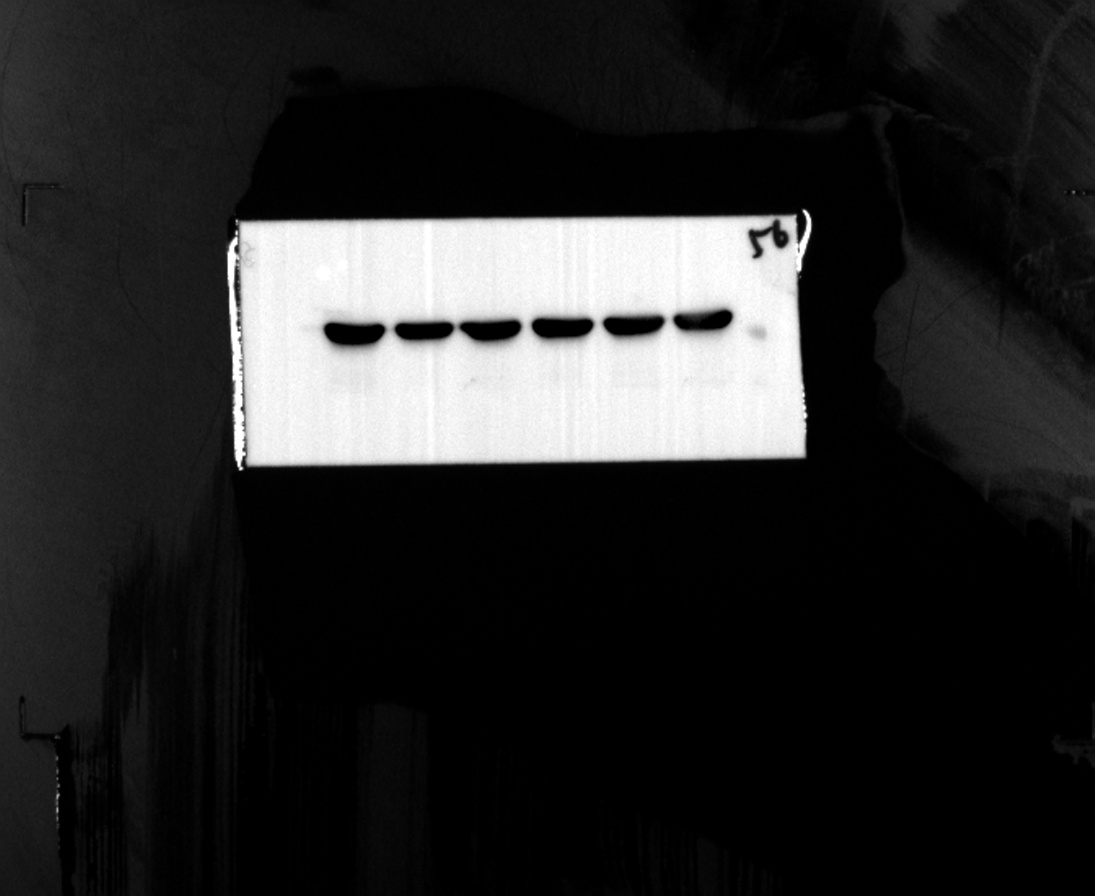




β-actin (Figure 7) β-actin (Figure 8)

**2. Original Western Blot images *in vivo***


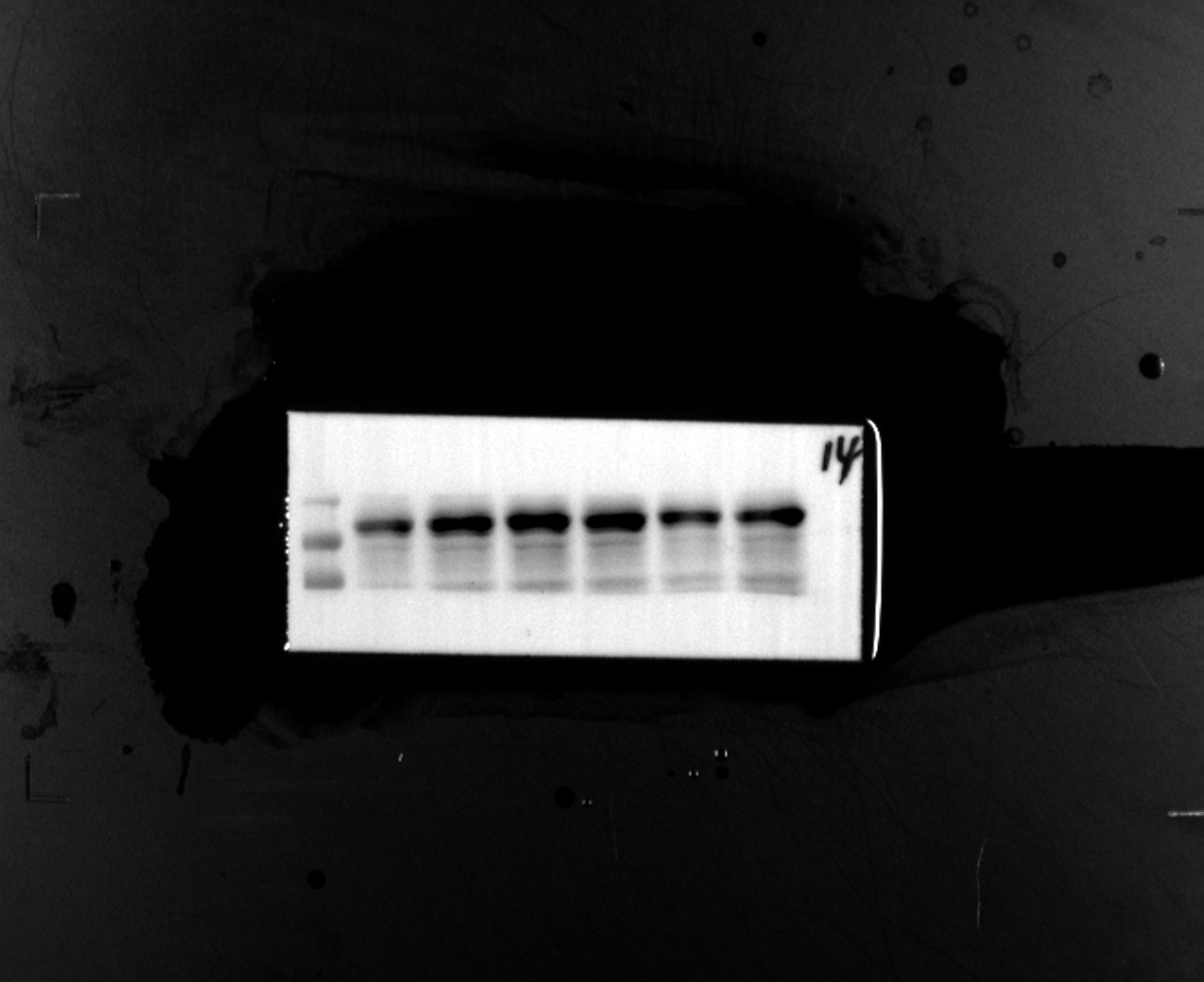

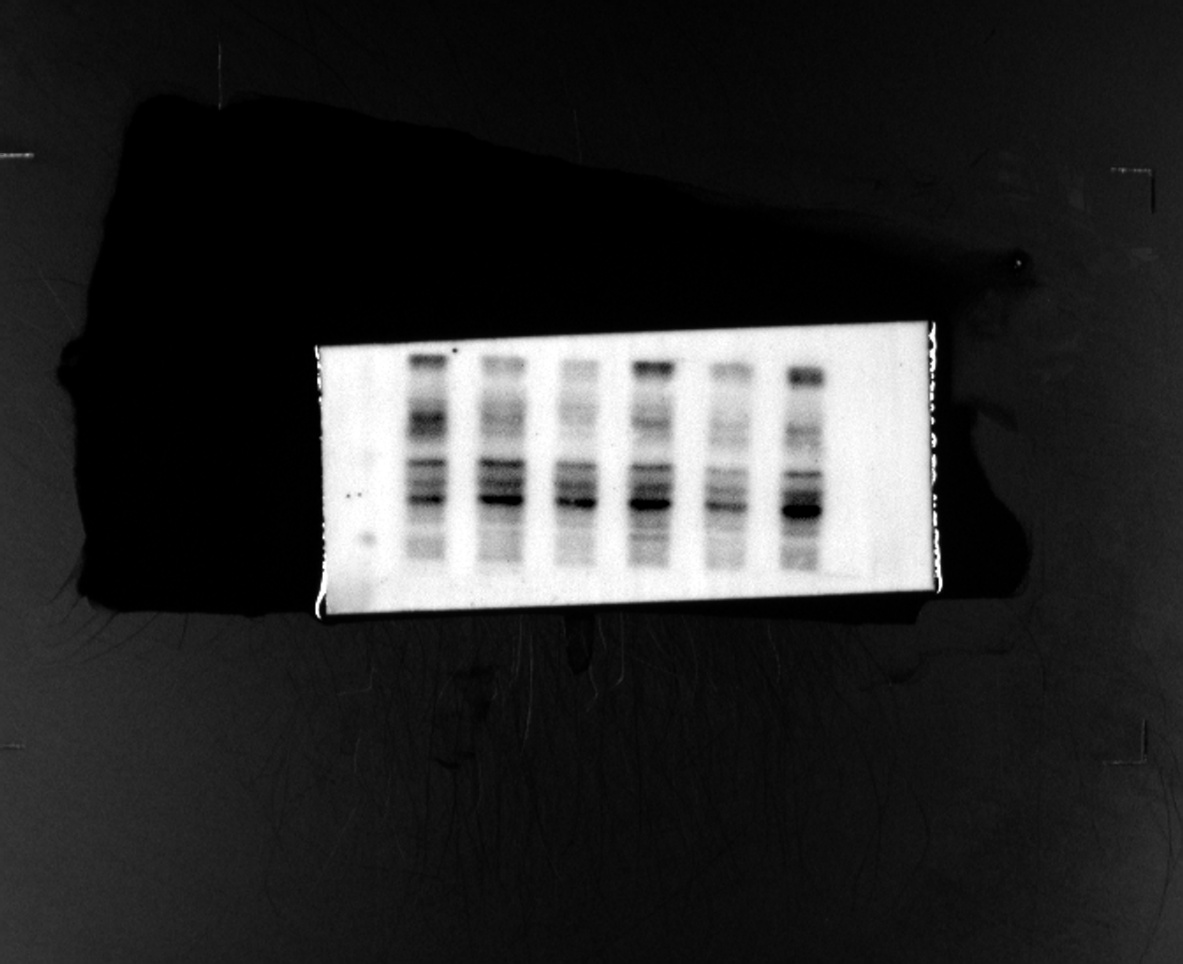


Csnk1g3-85aa IL-1β


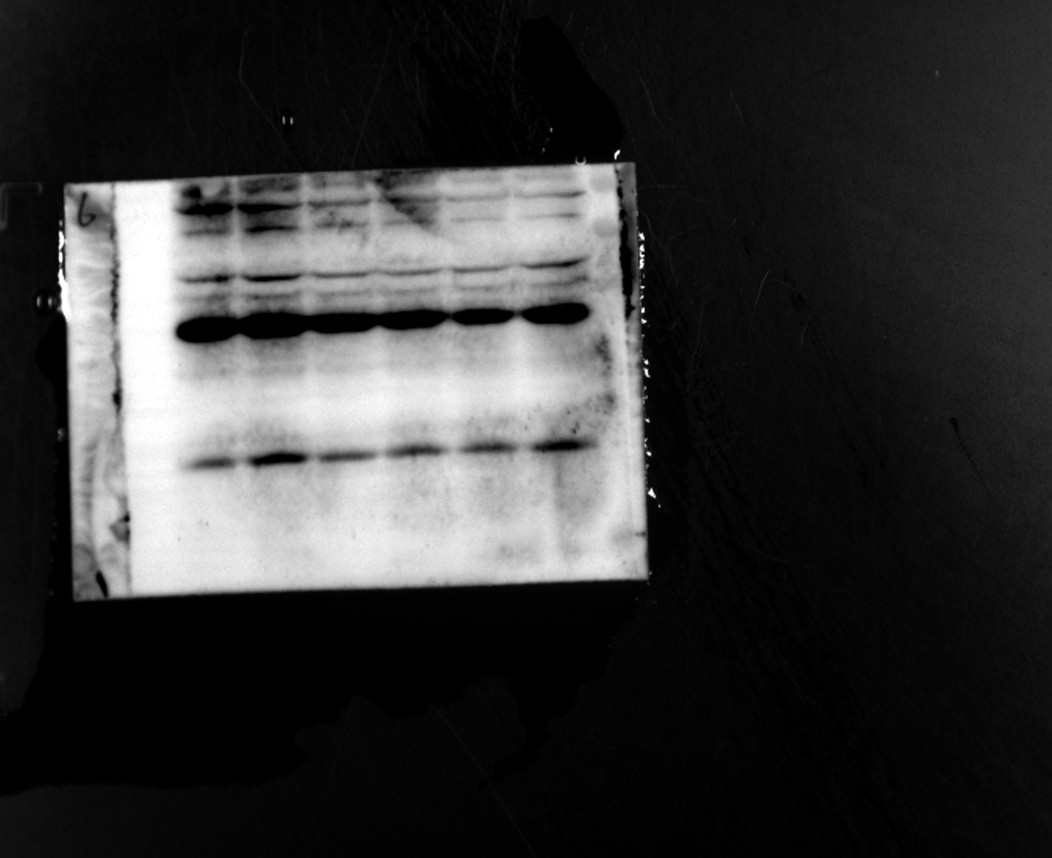

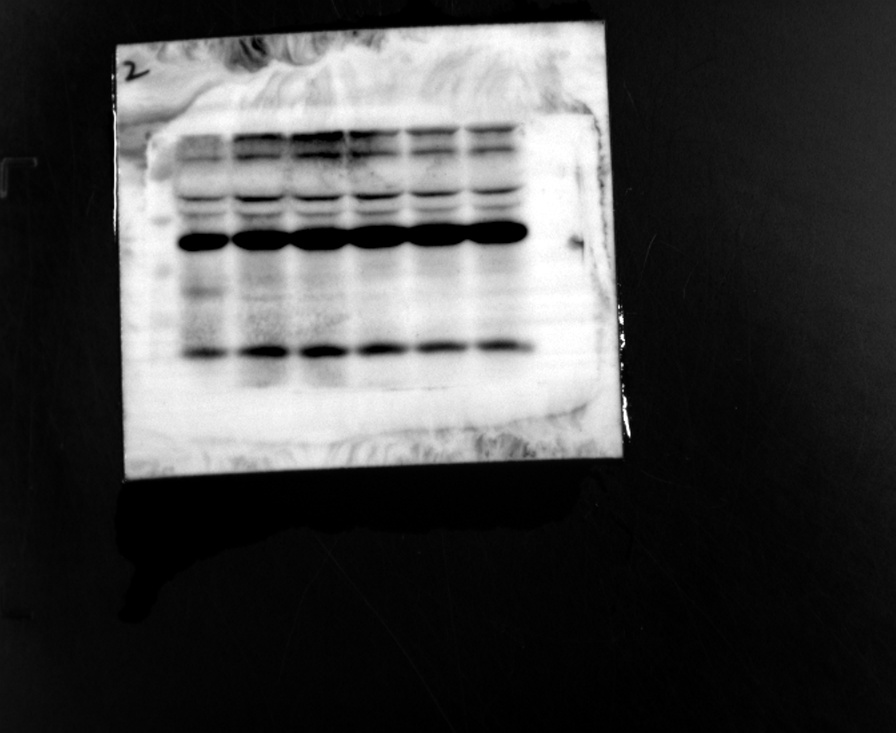


IL-6, CK1γ3 MLKL


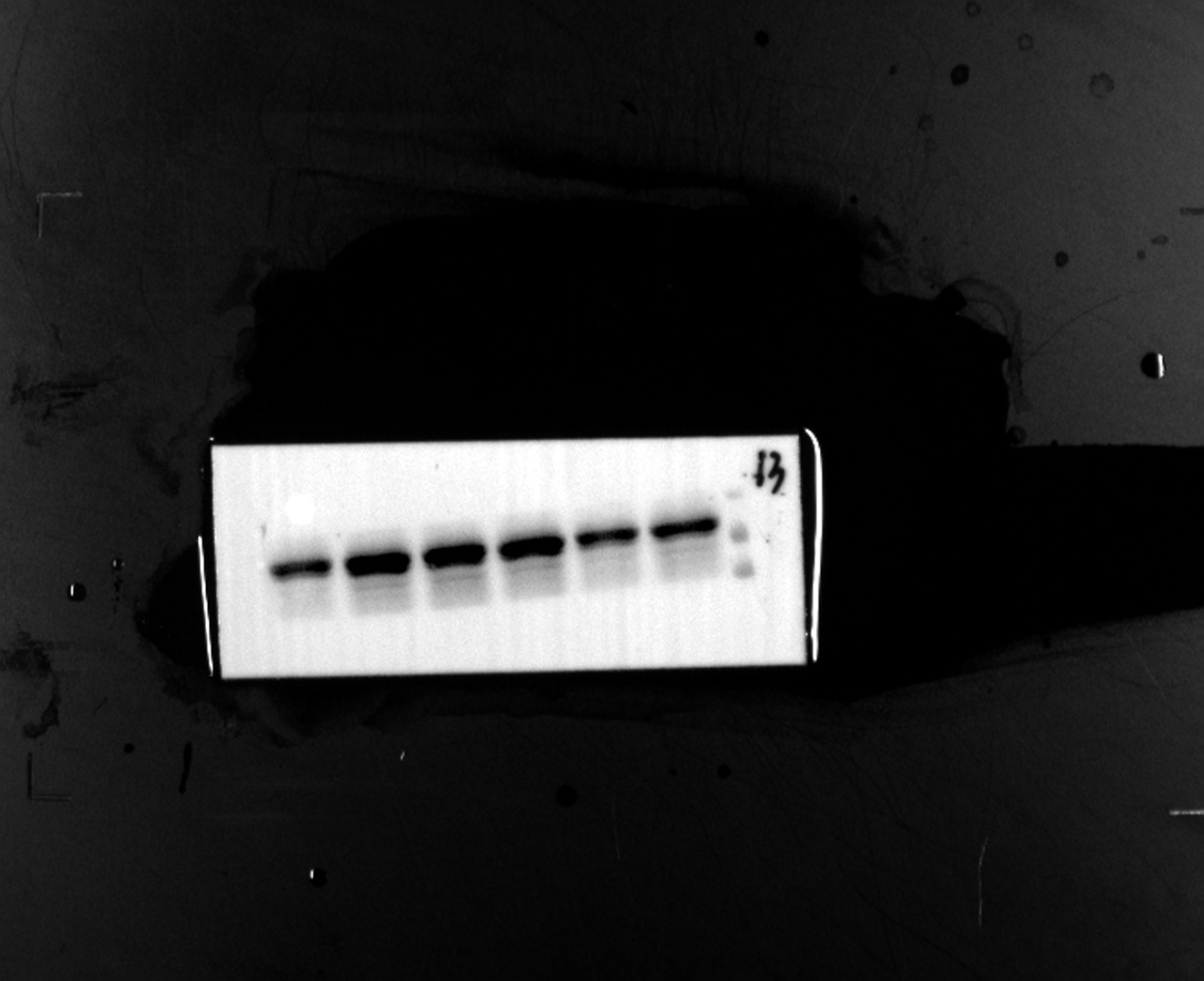

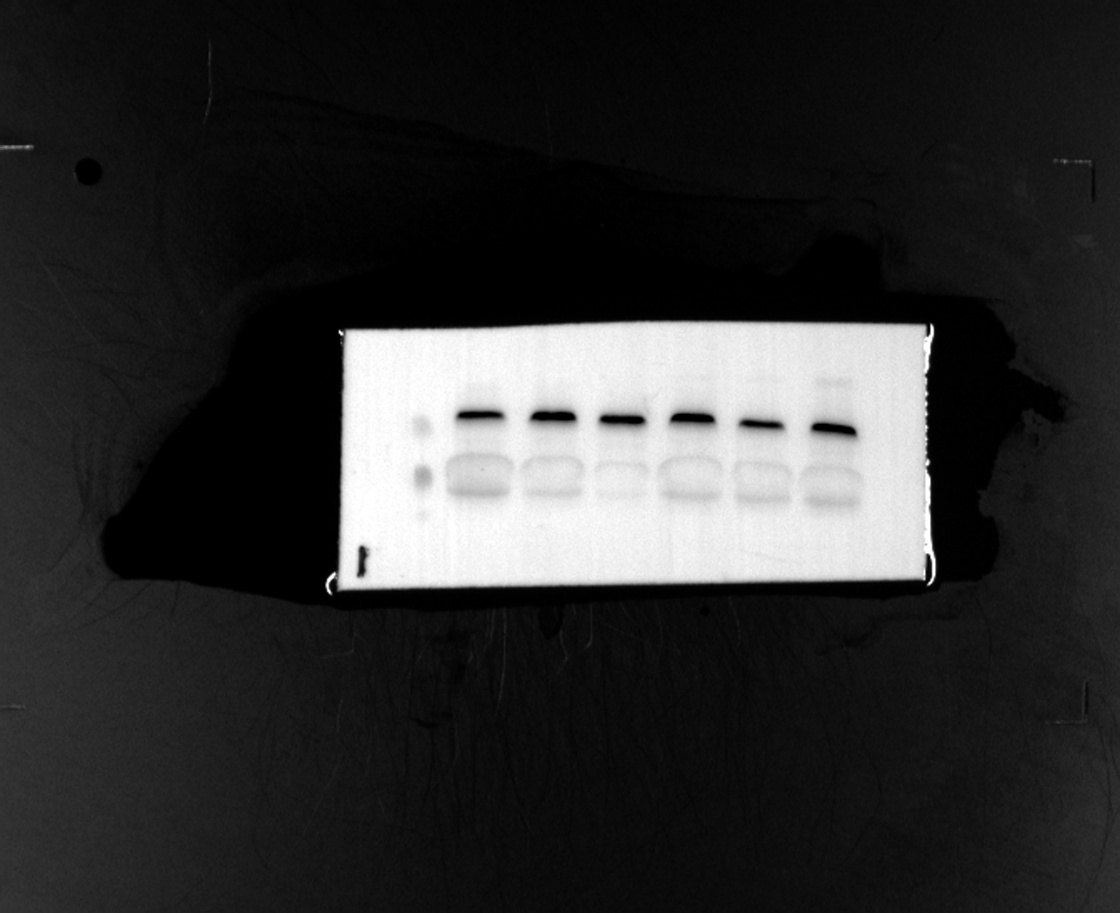


RIPK1 RIPK3


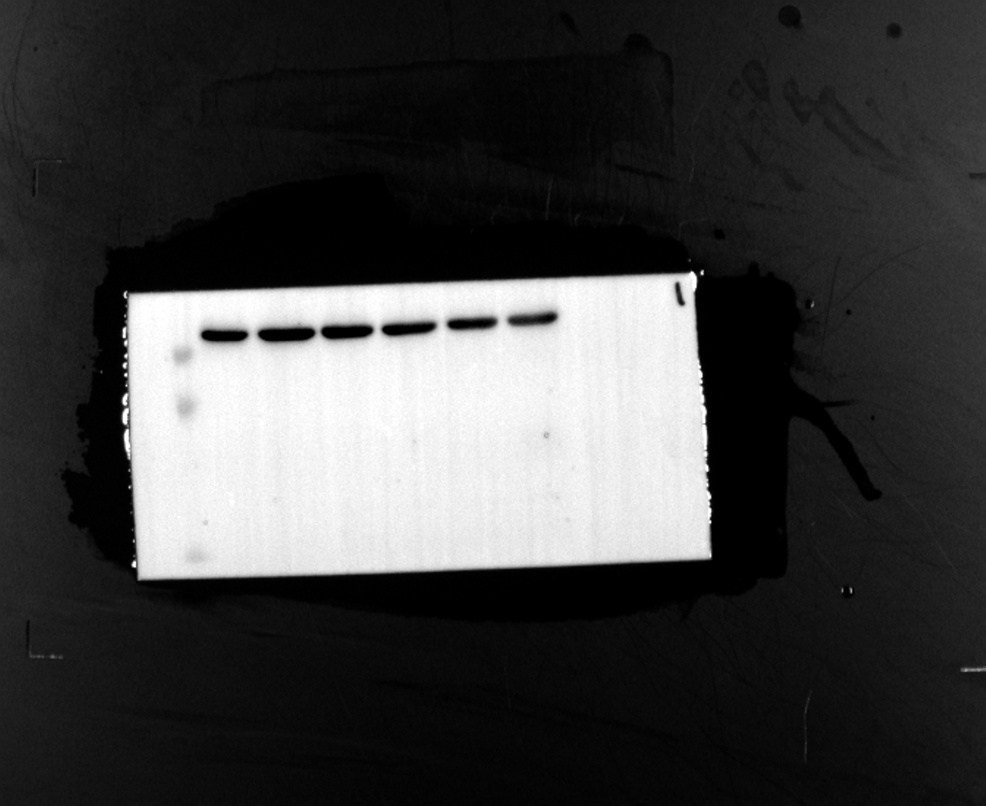

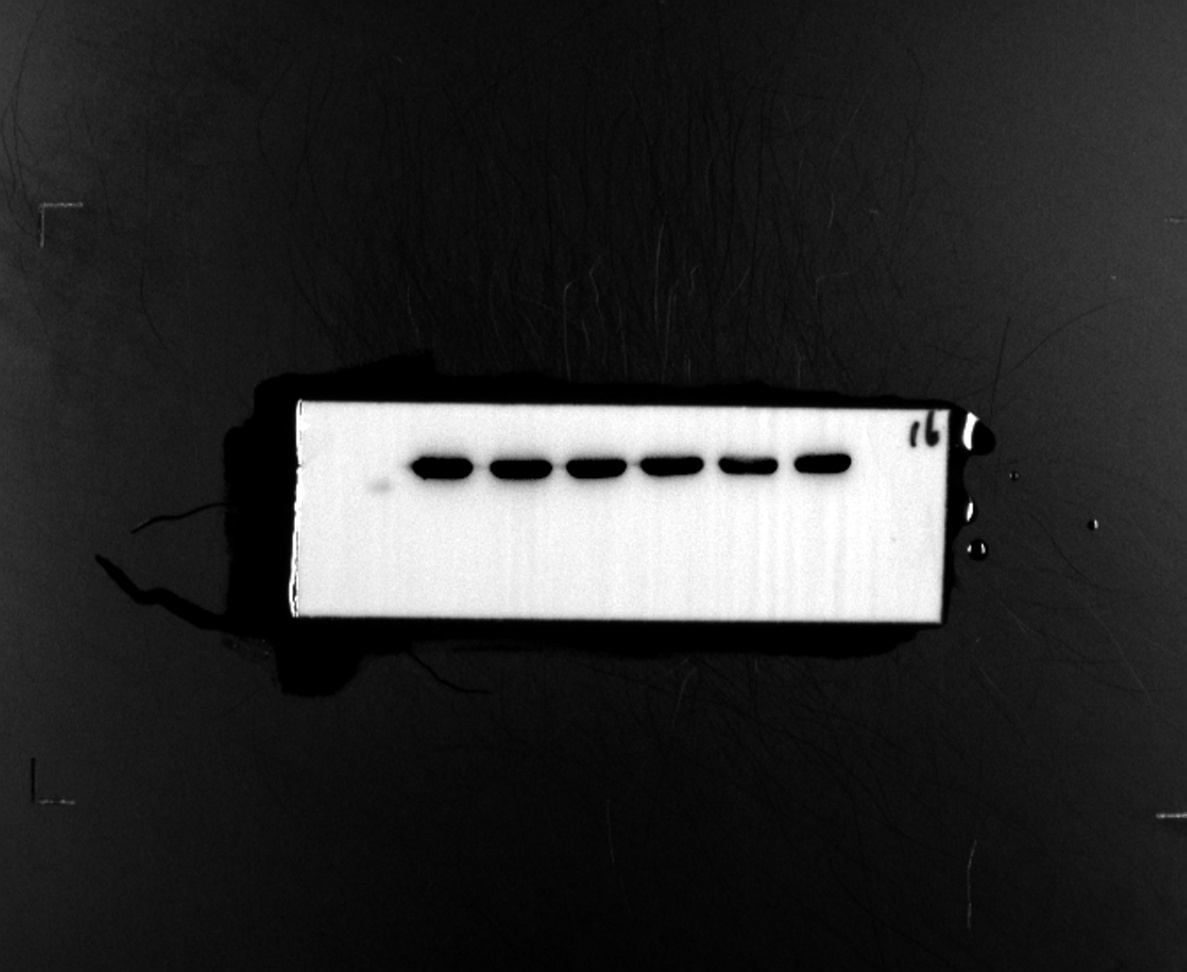


TNF-α β-actin (Figure 14)





β-actin (Figure 15)
